# Iron Deficiency Impairs Muscle Stem Cell Proliferation and Skeletal Muscle Regeneration via HIF‐2α Stabilization

**DOI:** 10.1002/jcsm.70124

**Published:** 2025-11-25

**Authors:** Wenyan Fu, Yang Liu, Amelia Yin, Liwei Xie, Hang Yin

**Affiliations:** ^1^ Center for Molecular Medicine The University of Georgia Athens Georgia USA; ^2^ Department of Biochemistry and Molecular Biology The University of Georgia Athens Georgia USA

**Keywords:** cell cycle regulation, HIF‐2α, iron deficiency, muscle regeneration, muscle stem cells, sarcopenia

## Abstract

**Background:**

Functional iron deficiency affects a large proportion of patients with chronic diseases and is increasingly observed in older adults. Clinical evidence links iron deficiency to sarcopenia, yet the mechanistic relationship between iron status and muscle regeneration remains poorly defined. This study investigates how iron depletion alters muscle stem cell (MuSC) proliferation and skeletal muscle regeneration, focusing on HIF‐2α signalling.

**Methods:**

Male and female *C57BL/6 J* mice (4 week old, *n* > 20 per group in total) were fed iron‐sufficient (IS) or iron‐deficient (ID) chow for 4 weeks before cardiotoxin‐induced tibialis anterior (TA) muscle injury. Muscle mass, MuSC proliferation and histological changes in regenerating TA muscles were evaluated at 10 and 30 days after injury (dpi). Pharmacological HIF‐2α inhibition (PT2385) was used to determine causal mechanisms. Data were analyzed by *t* tests and one‐way ANOVA.

**Results:**

Iron deficiency significantly reduced MuSC proliferation (−10.2% Ki67^+^ MuSC at 10 dpi, *p* < 0.01, *n* = 5) and myoblast EdU incorporation (−18.1%, *p* < 0.001), leading to smaller regenerating myofibres (−22.7% median cross‐sectional area at 30 dpi, *p* < 0.01, *n* = 3) and impaired muscle mass recovery (males: −13.9% *p* < 0.001, females: −9.4% *p* < 0.05, *n* = 6). HIF‐2α inhibition with PT2385 in ID mice increased MuSC proliferation (+7.1% Ki67^+^ MuSC at 10 dpi, *p* < 0.01, *n* = 5) and restored muscle mass (males: +10.3% *p* < 0.001, females: +5.5% *p* < 0.05, *n* = 6). Mechanistically, iron deficiency stabilized HIF‐2α in proliferating MuSC, which upregulated retinoblastoma protein (Rb1), repressed E2F target RNA levels and induced G0/G1 cell cycle arrest. This impaired myoblast expansion and delayed muscle regeneration in vitro and in vivo. In ID mice, PT2385 restored MuSC proliferation, accelerated myofibre maturation and enhanced muscle mass recovery without compromising MuSC self‐renewal. Chromatin immunoprecipitation demonstrated HIF‐2α binding at the *Rb1* promoter, increasing Rb transcription and reducing H3K27 acetylation at E2F target loci.

**Conclusions:**

Iron deficiency impairs skeletal muscle regeneration by stabilizing HIF‐2α in MuSC, inducing Rb1 RNA expression, and repressing E2F‐dependent proliferation. Transient HIF‐2α inhibition rescues MuSC proliferation and muscle repair under iron‐deficient conditions, highlighting HIF‐2α as a potential therapeutic target to counteract sarcopenia in aging and chronic diseases.

## 1 Introduction

Iron plays a pivotal role in cellular metabolism, serving as a cofactor for multiple enzymes involved in oxygen transport, oxygen sensing, histone demethylation and energy production, all of which are critical for muscle function and integrity [1]. Iron deficiency is a widespread nutritional disorder, affecting approximately 25% of the global population, with significant health implications. Its prevalence is markedly elevated in older individuals and in patients with chronic diseases such as chronic obstructive pulmonary disease (COPD), congestive heart failure (CHF), chronic kidney disease (CKD) and cancer cachexia, where rates can exceed 30%–50% [2, 3, 4, 5, 6]. This heightened prevalence is primarily attributed to chronic inflammation, which induces functional iron deficiency by sequestering iron in storage forms, thereby reducing its bioavailability for cellular processes [7]. These chronic conditions are also inextricably linked to muscle atrophy and sarcopenia, raising an intriguing involvement of iron deficiency in sarcopenia [1, 8]. Ameliorating sarcopenia in chronic diseases is critical, as it improves the quality of life, enhances physical function and reduces healthcare burdens, particularly in aging populations and those with progressive illnesses. Understanding the molecular mechanisms linking iron deficiency to sarcopenia in the context of chronic diseases is thus essential to develop targeted therapies that address this interplay and mitigate its detrimental consequences.

The long‐term maintenance of muscle mass and strength depends on muscle stem cells (MuSCs) or satellite cells, which transition from quiescence to active proliferation and differentiation following muscle injury to facilitate tissue repair [9]. Recent studies have begun to uncover iron's contributions to muscle regeneration. Che et al. demonstrated that iron deficiency triggers ferritinophagy, leading to impaired myoblast differentiation through RNF20‐mediated histone H2B ubiquitination [10]. Additionally, Corna et al. showed that macrophages play a key role in iron recycling during early muscle regeneration, acquiring iron from damaged tissue and exporting it via ferroportin to support myogenic repair [11]. While these findings underscore iron's importance in myoblast differentiation and muscle regeneration, the precise mechanism by which iron influences myoblast proliferation—a critical step in muscle regeneration—remains unknown. Addressing this knowledge gap will help establish the mechanistic basis of iron deficiency‐induced muscle dysfunction and identify potential therapeutic targets.

Iron deficiency in chronic diseases, cancer cachexia and aging not only diminishes heme and iron–sulfur (Fe–S) cluster synthesis but also reduces the availability of free iron (chelatable iron) [12]. Chronic kidney disease and chronic heart disease demonstrate a significant reduction in free iron [13, 14]. Free iron, particularly in its ferrous form (Fe^2+^), is vital for numerous biological functions, serving as a cofactor for mono‐ and dioxygenases, which regulate hypoxia responses, lipid peroxidation and histone demethylation. Among these, Fe^2+^ availability directly regulates hypoxia‐inducible factors (HIFs) through prolyl hydroxylase activity, linking iron status to oxygen‐sensing pathways. Hypoxia‐inducible factor 2α (HIF‐2α), in particular, is highly expressed in quiescent MuSC and decreases as MuSC re‐enter the cell cycle during activation. Stabilization of HIF‐2α impairs myoblast proliferation and muscle regeneration, as demonstrated in our prior work [15, 16]. Consistently, experimental hypoxia itself negatively impacts MuSC proliferation and delays muscle regeneration [17], and chronic hypoxia such as in COPD is associated with muscle atrophy and regenerative deficits [18]. These findings underscore the convergence of iron deficiency and hypoxia on HIF‐2α signalling in regulating MuSC function. Despite these established functions, the specific contribution of free Fe^2+^ to MuSC biology remains unclear. In particular, it is not known whether iron deficiency impairs MuSC proliferation and regenerative capacity by altering hypoxia‐responsive pathways or cell cycle regulation. Given the high prevalence of iron deficiency in aging and chronic diseases, we hypothesize that the chelatable free iron pool is essential for MuSC expansion and muscle repair. Here, we investigate how depletion of this pool affects MuSC proliferation and regeneration, with a focus on HIF‐2α as a potential iron‐sensitive regulator of stem cell dynamics.

## 2 Methods

### 2.1 Animal Housing and Strains

All animal studies were approved by the University of Georgia IACUC and performed following the guidelines. Animals were housed in a temperature‐controlled (22°C) environment with 12:12‐h light–dark cycles. The following mouse strains were used in this study, all obtained from the Jackson Laboratory: *C57BL6/J* (#000664), *Pax7* ^*creERT2*^ (#017763) and *R26R* ^*CAG‐Sun1/sfGFP*^ (#021039). Equal numbers of males and females were used in all experiments. To induce Cre activity in mice carrying *Pax7* ^*creERT2*^ allele, tamoxifen (20 mg/mL in corn oil, Sigma #C8267) was administered intraperitoneally for three consecutive days at a dosage of 100 mg/kg/day.

For iron sufficient (IS, 35 ppm, Dytes #115127) and iron deficient (ID, 3 ppm, Dytes #115072) chow feeding, 4‐week‐old *C57BL6/J* mice were housed in plastic microisolator cages with raised floor beds. Haemoglobin levels were measured using tail‐vein blood after 8‐week feeding to confirm iron‐deficient states by HemoCue Hb201 + Haemoglobin analyser (HemoCue).

### 2.2 Intramuscular Injections and Drug Treatments

To induce muscle injury, cardiotoxin (CTX; Sigma #C3987) was first reconstituted with sterile PBS (no Ca^2+^/Mg^2+^) and then injected into the tibialis anterior (TA) muscle of adult mice (0.5 nmol in 50 μL PBS). All injections were performed under general anaesthesia induced by 2–3% isoflurane delivered via a precision vaporizer, with mice maintained on a heated pad to prevent hypothermia. For pharmacological inhibition, PT2385 (1 μg in 50 μL 0.1% DMSO/PBS solution) or vehicle alone was injected into the TA muscle at 2, 3 and 4 days after injury (dpi), also under isoflurane anaesthesia.

### 2.3 Fluorescence Activated Cell Sorting and Cell Cycle Analysis

MuSC sorting was performed on a MoFlo XDP cell sorter (UGA CTEGD Cytometry Laboratory). GFP^+pos^ MuSC (~5 × 10^4^) were FACS‐sorted from 3‐month‐old *Pax7* ^*creERT2*^ *;R26R* ^*CAG‐Sun1/sfGFP*^ mice (at 1 week after tamoxifen induction). For cell cycle analysis, cells were stained with Hoechst 33342 (10 μg/mL) and analyzed on a HyperCyAn flow cytometer (UGA CTEGD Cytometry Laboratory). Cells of different cell cycle phases were calculated by FlowJo v10.

### 2.4 Cell Culture and Treatment

C2C12 myoblasts (CRL‐1772, ATCC) were cultured in DMEM (4.5 g/L glucose) medium supplemented with 10% FBS or 0.1% FBS (serum starvation) and 1% penicillin–streptomycin‐neomycin under 21% O_2_ and 5% CO_2_. For cell cycle manipulation, C2C12 myoblasts were serum‐starved in DMEM with 0.1% FBS for 48 h to induce quiescence (G0/G1 arrest), followed by refeeding with DMEM with 10% FBS for 72 h to promote cell cycle re‐entry. For Fe^2+^ depletion, 2,2′‐bipyridyl (BPD; 20 μM; Sigma Aldrich #D216305) were added to culture media.

### 2.5 Calcein AM Assay

Cell culture medium was removed, and cells were washed with PBS and then incubated with 0.25 μM Calcein AM (Life Technologies, C3100MP) in reaction buffer (4.5 g/L glucose, 2 mM glutamine in PBS) for 30 min at 37°C with 5% CO_2_. Then cells were washed twice and incubated in PBS for fluorescence intensity measurement at Ex/Em = 495/530 nm using CytoFlex Cytometer (Beckman Coulter) or fluorescence microscopy.

### 2.6 Cell Viability Assay

Cells were stained with ReadyProbe Cell viability imaging kit (Green and Blue, ThermoFisherScientific) under the guidance of manual instruction. Briefly, add two drops of NucGreen Dead reagent and two drops of NucBlue Live reagent per mL cell culture medium and incubate for 20 min at 37°C with 5% CO_2_. Fluorescence images were captured by EVOS fluorescence microscope (FITC/GFP filter for dead cells and DAPI filter for live cells).

### 2.7 Cell Counting and Proliferation Assay

For EdU staining, 10 μM EdU was added into the culture medium 4 h before assay. EdU Click‐It kit (ThermoFisherScientific #C10340) was utilized for the detection of the EdU signal.

For cell number counting, Hoechst 33342‐stained or EdU‐stained C2C12 myoblasts were counted by BioTek Lionheart FX automated microscope.

### 2.8 Muscle Histology Analyses

For immunostaining, TA muscle sections were fixed with paraformaldehyde (1% in PBS, 10 mins), permeabilized with Triton X‐100 (0.5%, 10 mins), blocked with Mouse on Mouse Blocking Reagent (Vector Lab) and 5% BSA/5% normal goat serum/PBS and incubated with primary antibodies: anti‐Pax7 (1:5; DSHB #Pax7), anti‐HIF‐2α (1:250; ThermoFisherScientific #PA‐16510), anti‐Ki67 (1:1000; Abcam #ab15580), anti‐Myogenin (1:50; DSHB #F5D), anti‐Laminin B2 (1:1000; Millipore #05–206), anti‐embryonic MyHC (eMyHC, 1:50, DSHB #F1.652) overnight at 4°C. Sections were washed in PBS/0.1% Tween‐20, incubated with AlexaFluor‐labelled secondary antibodies (1:200, 1 h), washed and mounted with DAPI‐containing mounting medium (Life Technologies). Mounted slides were imaged on a Zeiss LSM 710 confocal microscope.

For quantification of quiescent MuSC, Pax7^+^ nuclei were counted manually per cross‐section of uninjured muscle.

H/E staining was performed on muscle sections following an online protocol from the Treat‐NMD consortium (SOP: MDC1A_M.1.2.004).

The automated measurements of myofibre cross‐sectional areas on muscle IM images were performed as described in a previous publication (Mayeuf‐Louchart A. et al. *Skeletal Muscle* 2018).

### 2.9 eMyHC Quantification

Three non adjacent cryosections (~200 μm apart) were stained with anti‐eMyHC (DSHB F1.652, 1:50) together with anti‐Laminin B2 (1:1000) and DAPI. Images were acquired with identical exposure/gain across all groups. Quantification was performed in Fiji/ImageJ by an investigator blinded to group. Laminin B2 staining was used to generate a myofibre mask, defining total myofibre area. eMyHC signal was background‐subtracted and thresholded using an a priori fixed threshold determined from negative control images. Percents of eMyHC^+^ area were calculated as eMyHC‐positive pixels within the laminin mask divided by the total myofibre areas.

### 2.10 RNA‐Sequencing and Transcript Expression Calling

Total RNA was extracted from primary mouse myoblasts in three conditions: WT control, HIF‐2α overexpression (HIF‐2α OE) and HIF‐2α OE with HIF‐2α inhibition (100 nM PT2385). For each condition, three biological replicates were generated from separate time points using different founding myoblast isolates and clones. In each replicate, total RNA was isolated from two independently cultured wells and pooled to minimize intra‐replicate variability. To reduce sequencing costs while maintaining representative transcriptomic profiles, total RNA from the three biological replicates per condition was combined into a single composite sample for RNA‐seq library preparation. RNA was purified using RNA Extraction and Clean‐up kits (Zymo Research) and sequenced by BGI Genomics on the DNBSEQ platform.

### 2.11 Bioinformatic Analyses

RNA‐seq raw reads were first quality‐checked using FastQC. Cleaned reads were aligned to the mouse reference genome (GRCm38/mm10) using TopHat2. Following alignment, HTSeq‐count was used to generate the gene‐level count matrix based on RefSeq gene annotations. Normalization and differential expression analysis were performed using DESeq2 in R.

GSEA was performed using GSEA 4.0.3 (UC San Diego, Broad Institute) application with gene set databases: h.all.v2023.Hs.symbols.gmt [Hallmarks]. Gene sets were permutated 2000 times. Gene sets with normalized enrichment score (NES) ≥ 1 and a normalized *p* value **≤ 0**.05 were collected.

Unsupervised hierarchical clustering was performed using the online GenePattern platform (genepattern.org).

Rb1 promoter sequences were scanned for consensus hypoxia response elements (RCGTG) using the JASPAR database.

### 2.12 Statistics

Statistical analyses were conducted using Prism GraphPad 6.0/9.0 software. When specific pairs of groups are compared, the Shapiro–Wilk tests for normality (α = 0.05) were performed. If both groups follow normality, Two‐tailed Welch's *t* tests were conducted to test the equality of the means. When three or more than three groups are compared, one‐way ANOVA with Bonferroni's post hoc tests were employed to assess the significance of differences among multiple groups. The significant level (α) was set at 0.05. Data presented in figures represent mean ± SEM.

## 3 Results

### 3.1 MuSC Activation and Myoblast Re‐Entry Into Proliferation Are Associated With Increased Expression of Iron Uptake RNAs and Elevated Chelatable Iron Levels

**FIGURE 1:**
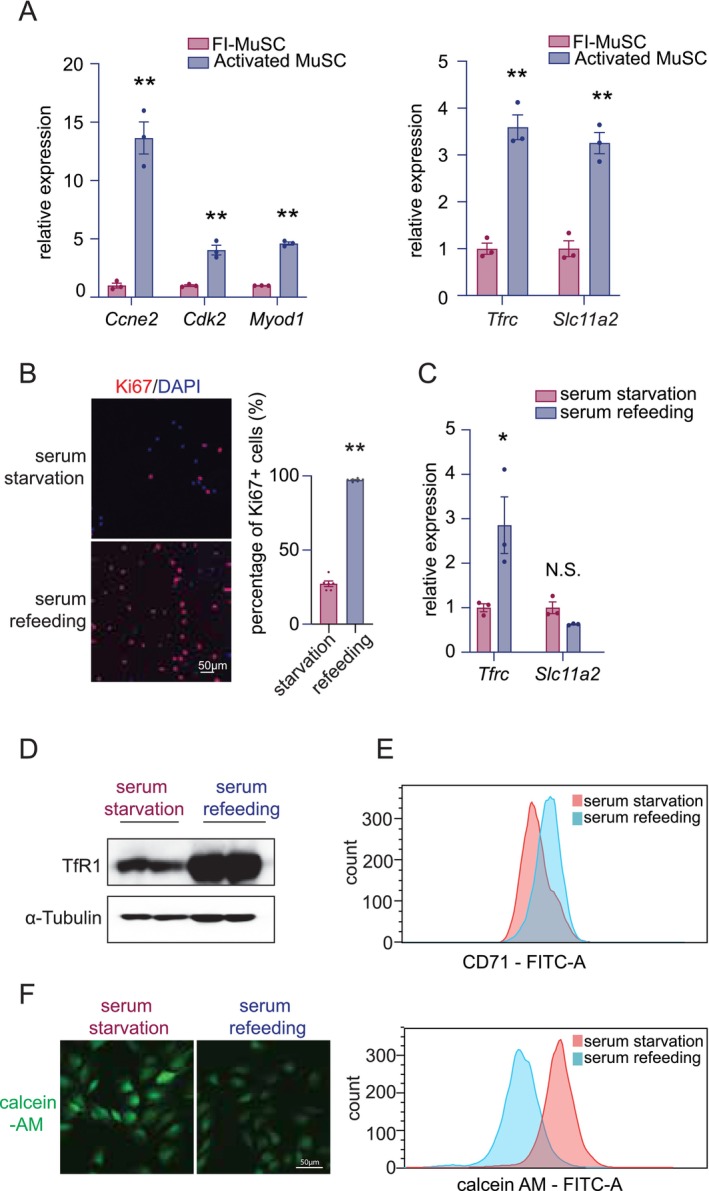
**Iron homeostasis‐related genes and intracellular iron levels are upregulated in activated MuSC and proliferating myoblasts. (A)** Left: RT‐qPCR analysis of proliferation‐related RNAs and *MyoD* RNA in MuSC isolated from uninjured muscle (FI‐MuSC) versus activated MuSC isolated from CTX‐injured muscle (5 dpi). Right: RT‐qPCR analysis of iron uptake‐related genes in FI‐MuSC versus activated MuSC. **(B)** Representative immunofluorescence staining of Ki67 (a proliferation marker) and DAPI in myoblast cultures. The ratio of Ki67^+^ myoblasts to all myoblasts indicates the proportion of proliferating cells. **(C)** RT‐qPCR analysis of iron uptake‐related genes in starved (noncycling) myoblasts versus refed (cycling) myoblasts. **(D)** Immunoblot showing higher TfR1 protein levels in cycling myoblasts compared to starved, noncycling myoblasts. α‐Tubulin serves as a loading control. **(E)** Flow cytometry analysis of cell‐surface CD71 (TfR1) in cycling myoblasts. **(F)** Representative images and flow cytometry histograms of calcein‐AM fluorescence demonstrating lower intracellular calcein‐AM signal in cycling myoblasts, indicative of higher intracellular iron content. Statistical comparisons were performed by Student's *t* tests. *p < 0.05, **p < 0.01, ***p < 0.001 and N.S. denotes not significant.

Iron importers transferrin receptor 1 (TfR1) and divalent metal transporter 1 (DMT1) are key regulators of intracellular iron homeostasis. We compared their expression levels in FACS‐isolated MuSC (FI‐MuSC) from uninjured TA muscle and activated MuSC from cardiotoxin (CTX)‐injured muscle at 5 days after injury (5 dpi). Activated MuSC exhibited elevated expression of cell cycle RNAs (*Ccne2*, *Cdk2*) and *Myod1* RNA>, and downregulation of quiescence‐associated genes (*Epas1*, *Spry1*), confirming their activation states (Figure 1A; Figure S1A). Notably, *Tfrc* (encoding TfR1) and *Slc11a2* (encoding DMT1) mRNA levels were significantly increased in activated MuSC compared to FI‐MuSC (Figure 1A).

We further examined their expression in C2C12 myoblasts under cell cycle arrest and serum refeeding‐induced proliferation states. Serum refeeding increased the proportion of proliferating cells from ~27% to ~97%, as indicated by fluorescence of cells with abundant Ki67 + proteins (Figure 1B). Importantly, this proliferative response occurred without induction of differentiation (Myogenin^−^) or cell death (PI^−^; Figure S1B–C). Compared to quiescent myoblasts, *Tfrc* mRNA was significantly elevated, whereas *Slc11a2* was unchanged in proliferating myoblasts (Figure 1C). Western blot confirmed increased TfR1 protein following serum refeeding, and flow cytometry of live cells stained for CD71 (TfR1) showed elevated surface TfR1 in proliferating cells (Figure 1D–E). These findings indicate that *Tfrc* is dynamically regulated during myoblast activation, resulting in increased surface expression of TfR1 in proliferating myoblasts.

To assess whether increased TfR1 expression correlates with changes in intracellular chelatable iron, we quantified intracellular chelatable Fe^2+^ using calcein‐AM staining (Supplemental Material—Ref. S1). Because Fe^2+^ quenches calcein fluorescence, a reduced signal indicates a higher free Fe^2+^ level. FACS analysis revealed significantly lower calcein‐AM fluorescence in proliferating myoblasts, consistent with elevated chelatable Fe^2+^ (Figure 1F).

### 3.2 Iron Depletion Impairs Myoblast Proliferation In Vitro and In Vivo

**FIGURE 2:**
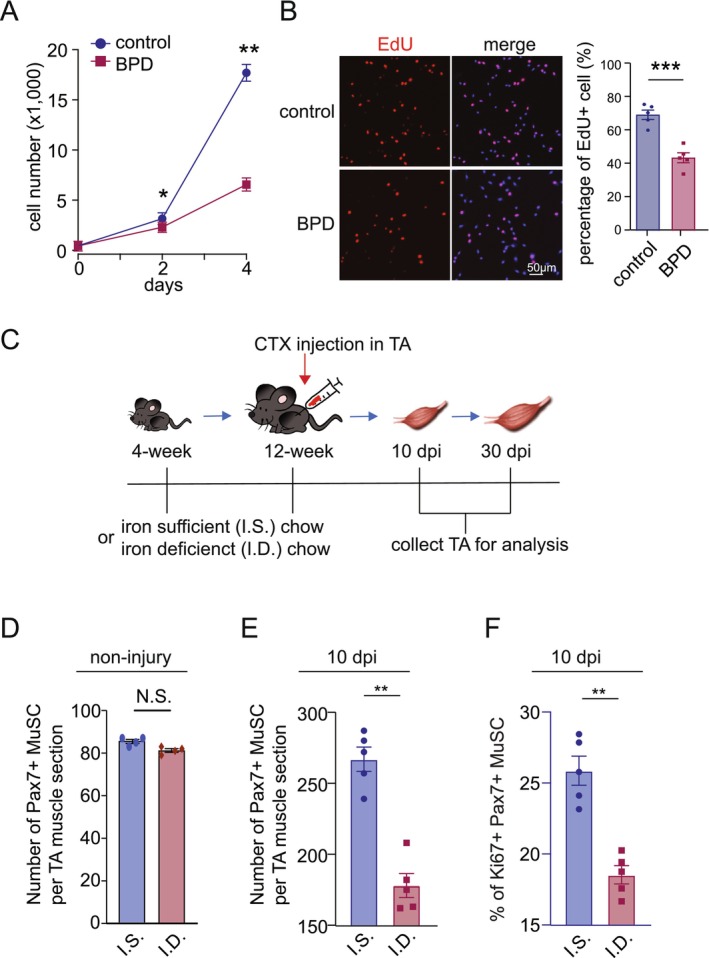
**Iron depletion impairs myoblast proliferation. (A)** Proliferation curves of control (DMSO) versus BPD‐treated C2C12 myoblasts over time. **(B)** Representative images of EdU labelling (red) and DAPI staining (blue) in control versus BPD‐treated C2C12 myoblasts. The right graph shows the percentage of EdU^+^ cells relative to total DAPI^+^ cells. **(C)** Schematic timeline depicting chow feeding regimens (iron‐sufficient vs. iron‐deficient diets), CTX injection and sample collection for downstream characterization. **(D)** Quantification of Pax7^+^ MuSC per TA muscle cross‐section in undamaged TA from IS and ID mice (*n* = 4). **(E)** Number of Pax7^+^ MuSC per TA muscle cross‐section at 10 dpi in IS and ID mice (*n* = 5). **(F)** The percentage of Pax7^+^/Ki67^+^ MuSC among total Pax7^+^ MuSC per TA muscle cross‐section at 10 dpi in IS versus ID mice (*n* = 5). All quantitative data represent mean ± SEM. Statistical comparisons were performed using Student's *t* test. *p < 0.05, **p < 0.01, ***p < 0.001 and N.S. denotes not significant.

To assess the role of chelatable Fe^2+^ iron in myoblast proliferation, we treated C2C12 myoblasts with the Fe^2+^ chelator 2,2′‐bipyridyl (BPD; Supplemental Material—Ref. S2) to model partial iron deprivation. BPD treatment for 5 days did not induce cell death (Figure S2A). However, proliferation assays showed that BPD‐treated myoblasts proliferate more slowly than controls, and EdU incorporation was significantly reduced (Figure 2A–B). These results suggest that Fe^2+^ depletion attenuates the proliferative capacity of myoblasts in vitro.

To examine the effect of iron depletion in vivo, we compared mice maintained on an iron‐deficient diet with those on an iron‐sufficient diet (Figure 2C). The number of quiescent Pax7^+^ MuSC per cross‐section was comparable between IS and ID in TA and soleus (Figure 2D, Figure S2B).

To assess regenerative proliferation, we induced muscle injury by CTX injection and analyzed TA muscles at 10 dpi. ID mice exhibited a significant reduction in total Pax7^+^ MuSC (Figure 2E). Moreover, co‐staining for Pax7 and Ki67 revealed a marked decrease in the proportion of proliferating (Pax7^+^/Ki67^+^) MuSC among total MuSC in ID mice compared to controls, indicating impaired MuSC activation and proliferation under iron‐deficient conditions (Figure 2F).

### 3.3 Iron Deficiency Impairs Skeletal Muscle Regeneration

**FIGURE 3:**
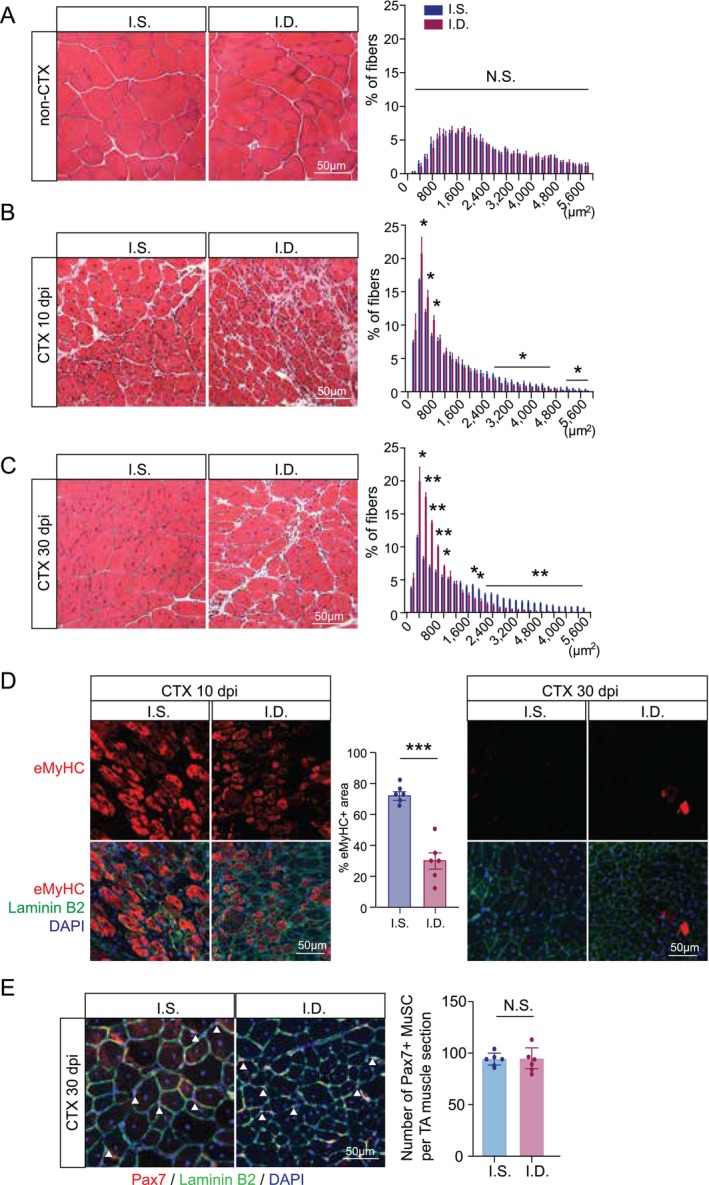
**Iron deficiency impairs skeletal muscle regeneration. (A)** Representative H&E staining and distribution of myofibre cross‐sectional areas in undamaged TA muscle from IS versus ID mice (*n* = 3). **(B)** Representative H&E staining and distribution of myofibre cross‐sectional areas in TA muscle at 10 dpi from IS versus ID mice (*n* = 3). **(C)** Representative H&E staining and distribution of myofibre cross‐sectional areas in TA muscle at 30 dpi from IS versus ID mice (*n* = 3). **(D)** Representative immunofluorescence images of embryonic myosin heavy chain (eMyHC, red) and Laminin B2 (green) in TA cross‐sections at 10 and 30 dpi from IS versus ID mice. Middle: quantification of percents of eMyHC^+^ areas at 10 dpi. **(E)** Representative images of Pax7 (red), Laminin B2 (green) and DAPI (blue) staining at 30 dpi in TA cross‐sections from IS versus ID mice, with quantification of Pax7^+^ MuSC per cross‐section (*n* = 6). All quantitative data represent mean ± SEM. Statistical comparisons were performed using Student's *t* test. *p < 0.05, **p < 0.01, ***p < 0.001 and N.S. denotes not significant.

To evaluate whether iron deficiency affects skeletal muscle regeneration, we collected TA muscles from IS and ID mice at 0, 10 and 30 dpi for histological analysis. After 8 weeks on the respective diets (0 dpi), TA muscle weights were significantly reduced in ID male mice while female mice showed a similar trend that did not reach statistical significance (Figure S3A). Body weights remained comparable between IS and ID groups (Figure S3B). At baseline (0 dpi), both groups showed normal muscle morphology, with well‐organized myofibres and peripheral myonuclei, and no notable differences in myofibre size (Figure 3A). A moderate reduction in haemoglobin levels, with unchanged haematocrit, confirmed a state of iron deficiency without anaemia in the ID mice (Figure S3C, S3D).

At 10 dpi, IS mice displayed robust regeneration with numerous newly formed myofibres containing centrally located nuclei—a hallmark of regeneration. In contrast, ID mice showed impaired regeneration, characterized by smaller myofibres, increased fibrotic areas and disorganized architecture (Figure 3B). By 30 dpi, IS muscles had largely returned to normal morphology, with mature, enlarged fibres and resolved inflammation. However, ID muscles still exhibited reduced fibre size, a higher proportion of small fibres (CSA ≤ 1200 μm^2^) and fewer large fibres (CSA ≥ 1800 μm^2^), along with persistent fibrotic areas, indicating delayed or incomplete regeneration (Figure 3C).

To further assess myofibre maturation, we stained for embryonic myosin heavy chain (eMyHC), a marker of nascent myofibres that is typically downregulated upon maturation. At 10 dpi, eMyHC^+^ fibres were abundant in IS muscles but relatively sparse in ID muscles, suggesting delayed differentiation. Quantification at 10 dpi confirmed a lower percent eMyHC^+^ area in ID versus IS (Figure 3D). By 30 dpi, eMyHC expression was nearly absent in IS muscles but remained in ID muscles, consistent with prolonged regeneration and delayed fibre maturation in the ID condition (Figure 3D).

To determine whether iron deficiency impacts MuSC self‐renewal, we stained for Pax7^+^ MuSC at 30 dpi. Pax7^+^ MuSC numbers were comparable between IS and ID groups, indicating that mild iron deficiency does not compromise MuSC maintenance after one round of regeneration (Figure 3E).

### 3.4 HIF‐2α Stabilization Under Iron Depletion Inhibits Myoblast Proliferation

**FIGURE 4:**
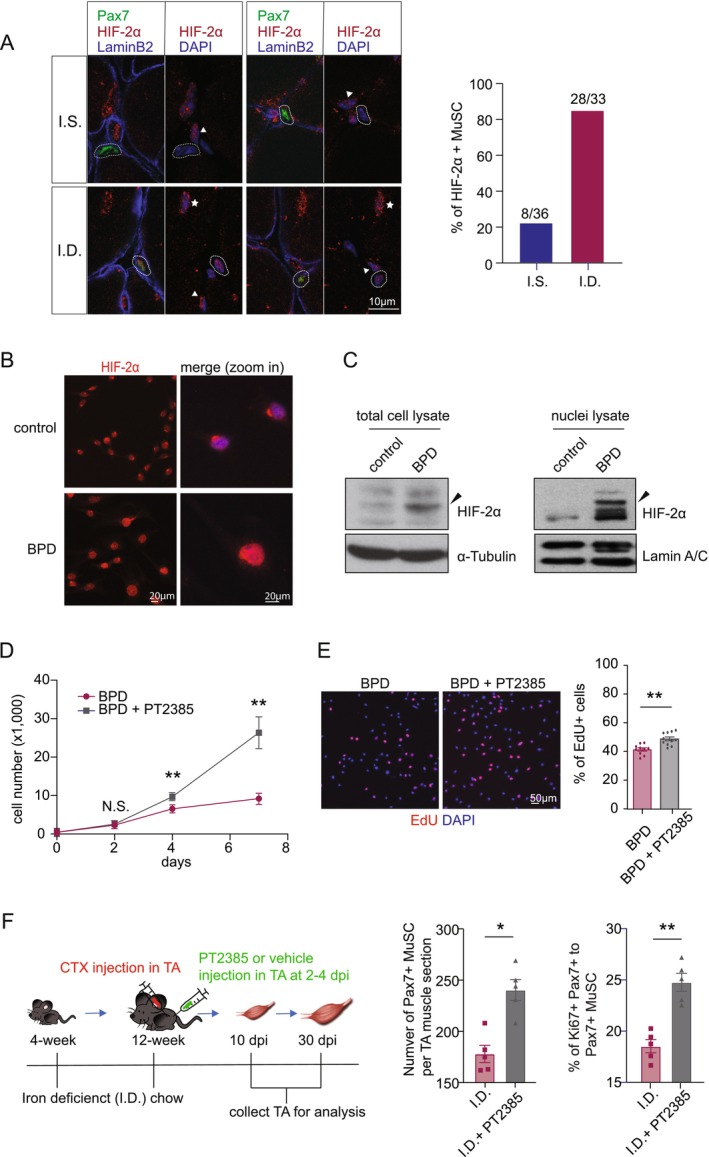
**Iron depletion–induced HIF‐2α stabilization blocks myoblast proliferation. (A)** Representative immunofluorescence images of HIF‐2α (red), Pax7 (green) and Laminin B2 (purple), along with DAPI staining, in TA muscle cross‐sections at 10 dpi from iron‐sufficient (IS) versus iron‐deficient (ID) mice. The bar graph (right) shows the proportion of HIF‐2α^+^ MuSC relative to total MuSC. **(B)** Representative images of C2C12 myoblasts (DAPI, blue) stained for HIF‐2α (red) under control (DMSO) versus BPD‐treated conditions. **(C)** Western blot analysis showing elevated levels of HIF‐2α in both whole‐cell and nuclear fractions of BPD‐treated myoblasts. α‐Tubulin serves as a loading control. **(D)** Proliferation curves of myoblasts treated with BPD alone versus BPD and PT2385 in combination. **(E)** Representative EdU (red) and DAPI (blue) staining in BPD versus BPD + PT2385–treated myoblasts, with quantification of EdU^+^ cells as a percentage of total nuclei. **(F)** Left: schematic timeline of PT2385 administration in iron‐deficient mice following CTX‐induced muscle injury. **Middle:** quantification of Pax7^+^ MuSC per TA cross‐section at 10 dpi in IS and ID mice (*n* = 5). Right: percentage of Pax7^+^/Ki67^+^ MuSC among total Pax7^+^ MuSC per cross‐section at 10 dpi in IS and ID mice (*n* = 5). All quantitative data represent mean ± SEM. Statistical comparisons were performed using Student's *t* test. *p < 0.05, **p < 0.01, ***p < 0.001 and N.S. denotes not significant.

Free Fe^2+^ is a cofactor for Prolyl Hydroxylases that are responsible for HIF‐α degradation under normoxia. Our previous work showed that HIF‐2α stabilization impairs muscle regeneration by reducing MuSC proliferation [15, 16], prompting the hypothesis that HIF‐2α mediates the anti‐proliferative effects of iron deficiency. To test this, we first examined whether HIF‐2α is stabilized in MuSC under iron deficiency. At 10 dpi, we co‐stained TA muscle sections from IS and ID mice for Pax7 and HIF‐2α. HIF‐2α^+^ nuclei were observed in three cell types: mononuclear immune cells, myocytes and Pax7^+^ MuSC (Figure 4A). Both IS and ID muscles showed HIF‐2α expression in immune cells (white arrowheads), but only ID muscles showed prominent nuclear HIF‐2α accumulation in myonuclei (white stars). In MuSC, most Pax7^+^ cells in IS muscle were HIF‐2α^−^, consistent with our prior findings that HIF‐2α is downregulated during MuSC activation [15]. In contrast, ~84% of Pax7^+^ MuSC in ID muscle were HIF‐2α^+^, compared to ~22% in IS muscle (white circles), indicating strong stabilization of HIF‐2α in the MuSC population under iron deficiency.

Consistent results were also observed in vitro. In C2C12 myoblasts, BPD treatment induced nuclear accumulation of HIF‐2α, whereas vehicle‐treated cells showed minimal peri‐nuclear staining (Figure 4B). Immunoblot analysis confirmed that both total and nuclear HIF‐2α levels were markedly increased following BPD treatment (Figure 4C).

To determine whether HIF‐2α mediates the proliferation block caused by iron depletion, we co‐treated BPD‐exposed myoblasts with the HIF‐2α–selective inhibitor PT2385. PT2385 significantly rescued the proliferation defect in BPD‐treated cells, as demonstrated by cell counts and restored EdU incorporation (Figure 4D–E). To validate this in vivo, we administered PT2385 intramuscularly in ID mice at 2–4 dpi following injury (Figure 4F). Treatment with PT2385 significantly increased the number of Pax7^+^ MuSC and the proportion of Ki67^+^ proliferating MuSC compared to vehicle‐treated ID controls, indicating that HIF‐2α is a critical mediator of the proliferation block caused by iron deficiency (Figure 4F).

### 3.5 HIF‐2α Represses E2F Target RNA Expression via Transcriptional Activation of Rb1

**FIGURE 5:**
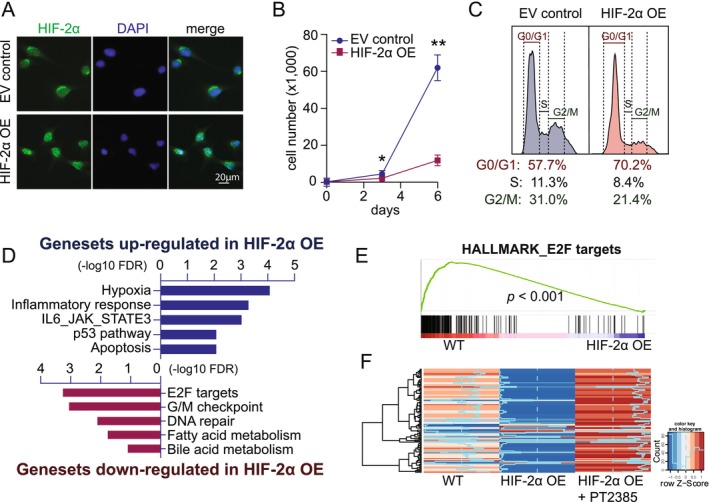
**HIF‐2α represses E2F target gene expression. (A)** Representative immunofluorescence images of HIF‐2α (green) and DAPI (blue) in wild‐type (WT) C2C12 myoblasts versus myoblasts overexpressing HIF‐2α (HIF‐2α OE) upon doxycycline (dox) induction. **(B)** Proliferation curves of WT versus HIF‐2α OE myoblasts. **(C)** Flow cytometry analysis of cell‐cycle distribution in WT versus HIF‐2α OE myoblasts. **(D)** Gene Set Enrichment Analysis (GSEA) highlighting gene sets significantly upregulated (blue) or downregulated (red) in HIF‐2α OE myoblasts. **(E)** GSEA enrichment plot for the *E2F_targets* gene set in HIF‐2α OE myoblasts. **(F)** Heatmap displaying the expression of E2F target RNAs in WT, HIF‐2α OE and HIF‐2α OE myoblasts treated with PT2385. Blue: low expression level; red: high expression level. Quantitative data represent mean ± SEM. Statistical comparisons were performed using Student's *t* test. *p < 0.05, **p < 0.01, ***p < 0.001 and N.S. denotes not significant.

To determine whether HIF‐2α is sufficient to inhibit myoblast proliferation, we overexpressed a stabilized triple‐mutant form of HIF‐2α (P405A/P530V/N851A, which is resistant to hydroxyproline‐induced degradation) in C2C12 myoblasts. HIF‐2α overexpression (OE) resulted in robust induction of nuclear HIF‐2α, as confirmed by immunofluorescence and immunoblotting (Figure 5A, Figure S4A). HIF‐2α OE myoblasts exhibited reduced proliferation and EdU incorporation compared to Dox‐treated control cells (infected with empty vector, EV), similar to BPD‐treated cells (Figure 5B, Figure S4B). Flow cytometry revealed a G0/G1 arrest in HIF‐2α OE cells, with an increased proportion of cells in G0/G1 (70.2%) compared to EV control cells (57.7%), suggesting that HIF‐2α inhibits proliferation by blocking G1/S transition (Figure 5C).

We next performed RNA‐seq comparing WT and HIF‐2α OE cells. As expected, canonical HIF‐2α targets such as *Atp7a*, *Cav1* and *Vegfa* were upregulated (Figure S4C). Gene Set Enrichment Analysis (GSEA) showed enrichment of hypoxia‐ and inflammation‐related pathways among transcripts with increased expression. Strikingly, the top downregulated gene set was **‘**E2F TARGETS**,’** which include transcripts encoding G1/S cell cycle regulators such as *Ccna2*, *Ccnb1* and *Cdk2* (Figure 5D–E). Hierarchical clustering and RT‐qPCR confirmed repression of E2F targets upon HIF‐2α OE, which was fully rescued by PT2385 co‐treatment (Figure 5F, 6A). Similarly, BPD treatment in normal C2C12 myoblasts reduced the expression of E2F target genes, which was partially rescued by PT2385, as evidenced by an increase of Ccna2 mRNA to ~45% and Ccnb1 mRNA to ~33% of control levels—both significantly higher than BPD alone (Figure S4D). This supports that HIF‐2α mediates the repression of E2F targets under iron deficiency.

**FIGURE 6:**
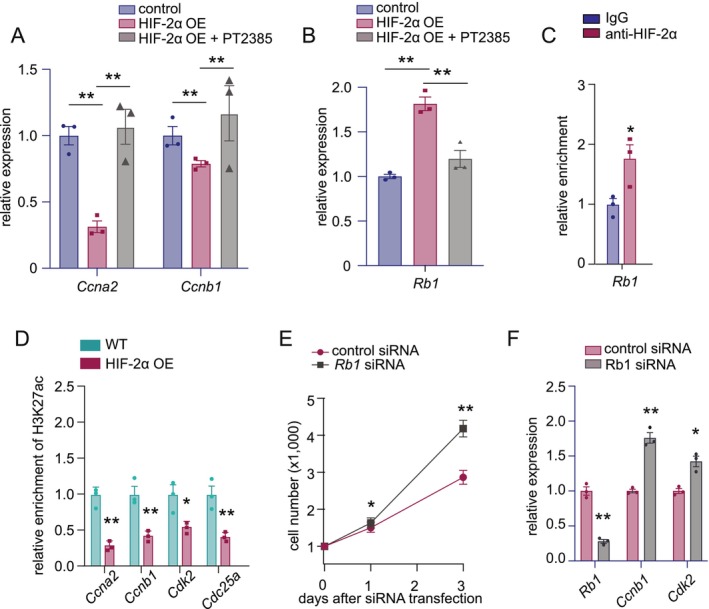
**HIF‐2α inhibition rescues E2F target RNA expression and HIF‐2α transactivates Rb1. (A)** RT‐qPCR analysis of representative E2F targets (*Ccna2* and *Ccnb1*) in WT versus HIF‐2α OE myoblasts, with or without PT2385 treatment. **(B)** RT‐qPCR analysis of *Rb1* mRNA in WT versus HIF‐2α OE myoblasts, with or without PT2385 treatment. **(C)** ChIP‐qPCR analysis of HIF‐2α occupancy in the HRE‐containing promoter region of *Rb1*. **(D)** ChIP‐qPCR analysis of H3K27ac occupancy in the promoter regions of representative E2F targets (*Ccna2*, *Ccnb1*, *Cdk2*, *Cdc25a*) in WT versus HIF‐2α OE myoblasts. **(E)** Proliferation curves of HIF‐2α OE myoblasts transfected with control siRNA versus *Rb1* siRNA. **(F)** RT‐qPCR analysis of *Rb1* and representative E2F targets (*Ccnb1* and *Cdk2*) in HIF‐2α OE myoblasts transfected with control siRNA versus *Rb1* siRNA. Quantitative data represent mean ± SEM. Statistical comparisons in (C), (D), (E) and (F) were performed using Student's *t* test. Statistical comparisons in (A) and (B) were performed using one‐way ANOVA with Tukey HSD. *p < 0.05, **p < 0.01, ***p < 0.001 and N.S. denotes not significant.

The Rb pathway is a well‐established regulator of E2F activity. We hypothesized that HIF‐2α represses E2F targets through Rb induction. Indeed, *Rb1* (encoding Rb) mRNA was significantly upregulated in HIF‐2α OE cells, and this induction was suppressed by PT2385 (Figure 6B). BPD‐treated myoblasts also showed elevated *Rb1* expression (Figure S5A). A conserved hypoxia response element (HRE) locates in the *Rb1* promoter (Figure S5B). Similar to known HIF‐2α targets *Vegfa* and *Cav1*, ChIP‐qPCR confirmed HIF‐2α binding to the *Rb1* promoter, supporting direct transcriptional regulation (Figure 6C, Figure S5C).

To investigate whether HIF‐2α represses E2F targets through Rb‐mediated chromatin remodelling [19, 20], we performed ChIP‐qPCR for the transcriptionally active histone mark H3K27ac at E2F target promoters. HIF‐2α OE significantly reduced H3K27ac levels at *Ccna2*, *Ccnb1*, *Cdk2* and *Cdc25a*, consistent with transcriptional repression (Figure 6D, Figure S5D). Finally, *Rb1* knockdown via siRNA reversed the proliferation defect in HIF‐2α OE cells and restored RNA expression of E2F targets, confirming that Rb is functionally required for HIF‐2α–mediated repression (Figure 6E–F).

Together, these results identify Rb as a direct transcriptional target of HIF‐2α and demonstrate that HIF‐2α suppresses myoblast proliferation by activating Rb and inhibiting E2F target RNA expression.

### 3.6 Transient HIF‐2α Inhibition Promotes Muscle Regeneration in Iron‐Deficient Mice

**FIGURE 7:**
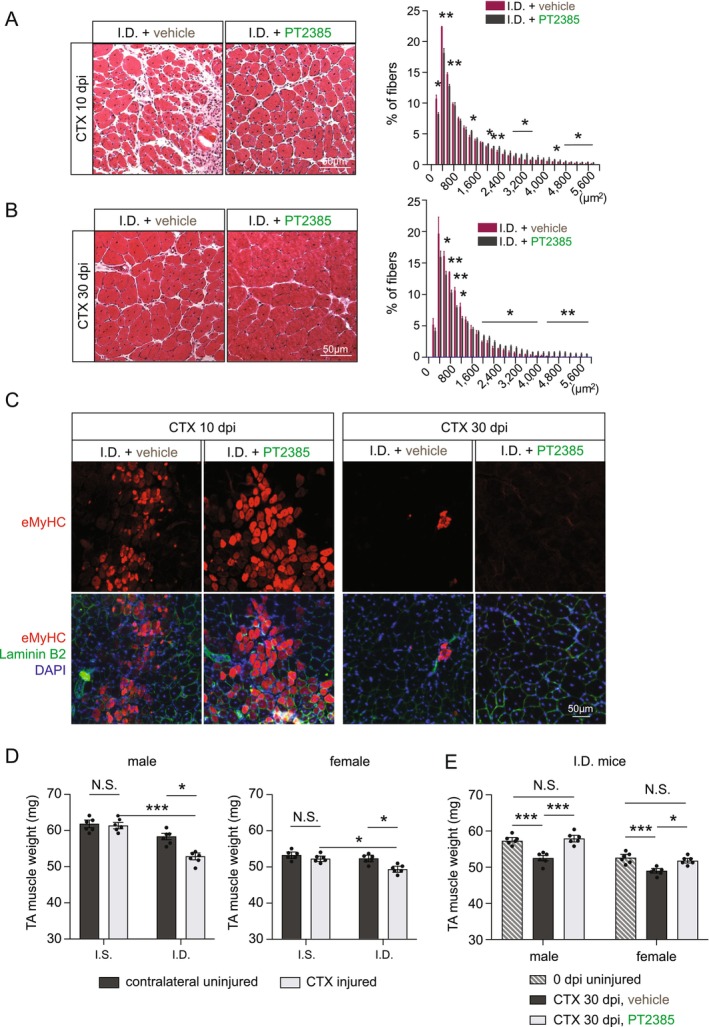
**Transient pharmacological HIF‐2α inhibition improves muscle regeneration under iron deficiency. (A)** Representative H&E‐stained TA muscle cross‐sections at 10 dpi from ID mice treated with either vehicle (0.1% DMSO) or PT2385, along with distributions of myofibre cross‐sectional area (*n* = 3). **(B)** Representative H&E‐stained TA muscle cross‐sections at 30 dpi from ID mice treated with vehicle or PT2385, including myofibre cross‐sectional area distributions (*n* = 3). **(C)** Representative immunofluorescence images of eMyHC (red) and Laminin B2 (green) in TA cross‐sections at 10 and 30 dpi from vehicle‐ or PT2385‐treated ID mice. **(D)** Weights of CTX‐injured TA muscles versus contralateral uninjured TA in IS versus ID mice at 30 dpi. (*n* = 6 per group) **(E)** Weights of CTX‐injured TA muscles at 30 dpi from ID mice treated with vehicle or PT2385, compared to uninjured TA at 0 dpi. (*n* = 6 per group) Quantitative data represent mean ± SEM. Statistical comparisons in (A) and (B) were performed using Student's *t* test. Statistical comparisons in (D) and (E) were performed using one‐way ANOVA with Tukey HSD. *p < 0.05, **p < 0.01, ***p < 0.001 and N.S. denotes not significant.

Given the central role of HIF‐2α in mediating proliferation arrest under iron‐deficient conditions, we tested whether transient pharmacological inhibition of HIF‐2α could improve muscle regeneration under iron deficiency. Following the same dosing schedule as described in Figure 4, we administered PT2385 directly into the TA muscle of ID mice after injury and collected tissue at 10 and 30 dpi for analysis. At both 10 dpi and 30 dpi, PT2385‐treated ID muscles exhibited larger regenerated myofibres compared to vehicle‐treated ID controls, indicating improved myofibre growth (Figure 7A–B). Immunostaining for eMyHC revealed enhanced expression at 10 dpi and near‐complete resolution by 30 dpi in PT2385‐treated muscles, suggesting that HIF‐2α inhibition accelerates myofibre maturation (Figure 7C). At 30 dpi, the weights of regenerated TA muscle were comparable with those from 0 dpi/uninjured states in the IS group; where a significant reduction was observed in the ID group (Figure 7D). Notably, PT2385 treatment at 2–4 dpi rescued the injury‐associated muscle mass loss in the ID group (Figure 7E). The number of Pax7^+^ MuSC at 30 dpi was comparable between PT2385‐treated and control ID groups, indicating that short‐term HIF‐2α inhibition does not affect MuSC self‐renewal (Figure S5A).

## 4 Discussion

In this study, we demonstrate that iron is essential for MuSC proliferation and that iron deficiency impairs MuSC expansion, leading to defective muscle repair. Mechanistically, we show that reduced iron level stabilizes HIF‐2α, which in turn induces Rb expression, represses E2F pathway and enforces cell cycle arrest. Strikingly, we find that pharmacological inhibition of HIF‐2α with PT2385 rescues muscle regeneration defects under iron‐deficient conditions, leading to a significant recovery of muscle mass.

While our data demonstrate that HIF‐2α directly regulates the Rb‐E2F axis in MuSC, we acknowledge that HIF‐2α also functions in other regenerative niche cell types, including macrophages and endothelial cells. Although our intramuscular PT2385 delivery suggests a MuSC‐intrinsic effect, future studies using MuSC‐specific HIF‐2α knockout models and examining the interplay between MuSC and immune cells will fully elucidate the role of HIF‐2α in muscle regeneration under iron‐deficient conditions. Furthermore, although we observed robust upregulation of TfR1 and free iron accumulation in proliferating myoblasts, we were unable to directly quantify free iron levels in MuSC in vivo due to technical limitations. Finally, as our study was conducted in young mice, it will be important to validate this mechanism in aged or disease‐relevant models with co‐occurring iron deficiency and sarcopenia in future studies.

Our finding that both acute Fe^2+^ depletion and chronic iron deficiency induce HIF‐2α in MuSC is consistent with previous observations in other cell types [21, 22]. The regulation of HIF‐2α by iron metabolism occurs at multiple levels: 1) Fe^2+^ is a key cofactor for prolyl hydroxylase domain enzymes, which regulates HIF‐2α degradation, 2) Fe–S clusters regulate HIF‐2α translation via IRP2, acting as a posttranscriptional checkpoint [23]; and 3) heme, a potential cofactor for HIF‐2α's PAS‐B domain, may influence its nuclear localization [24]. Conversely, HIF‐2α also regulates systemic iron homeostasis, facilitating iron release from liver stores [21] and increasing intestinal iron absorption via duodenal epithelial cells [25]. Additionally, HIF‐2α promotes erythropoiesis by upregulating EPO expression [26], linking its iron sensor activity to safeguard of oxygen delivery to vital organs through iron redistribution. Notably, studies have shown that iron redistribution under deficiency states is tissue‐specific—for instance, while cardiac myoglobin levels remain stable under iron deficiency, gastrocnemius muscle myoglobin decreases by 35% [27], highlighting the complexity of iron prioritization among tissues in deficient states.

The RB‐E2F pathway is a highly conserved regulator of cell‐cycle progression, controlling G1/S transition. Both HIF‐2α and Rb are highly expressed in quiescent stem cell populations, including MuSC in uninjured muscle [15, 28, 29, 30]. Interestingly, genetic ablation of either HIF‐2α or Rb in quiescent MuSC leads to premature cell cycle entry in the absence of tissue damage signals [15, 28], suggesting that HIF‐2α and Rb function together to maintain MuSC quiescence. During MuSC activation, Rb undergoes CDK‐mediated hyperphosphorylation, releasing E2F transcription factors to drive S‐phase entry [28]. Our previous work showed that HIF‐2α expression acutely declines during early MuSC activation, preceding cell cycle entry [15], suggesting that Rb inactivation during MuSC activation is regulated both transcriptionally and posttranslationally.

At the early stages of muscle regeneration, efficient MuSC proliferation is essential for later myonuclear accretion, muscle volume restoration and suppression of pro‐inflammatory/pro‐fibrotic signalling [9]. We previously reported that HIF‐2α stabilization under chronic hypoxia suppresses MuSC proliferation, contributing to sarcopenia [16]. The current study extends this finding, revealing that iron deficiency also stabilizes HIF‐2α and activates the HIF‐2α/Rb axis in proliferating MuSC/myoblasts, suggesting a direct role of iron deficiency in long‐term muscle health and sarcopenia in chronic diseases, cancer cachexia and aging.

Beyond its regulation by hypoxia and iron, accumulating evidence suggests that HIF‐2α activity is influenced by oxidative stress (affecting Fe^2+^/Fe^3+^ ratio and mTORC2 activity, which regulates HIF‐2α translation) and inflammation [31, 32, 33]. Given the high prevalence of these stressors in aging and chronic diseases, it is plausible that HIF‐2α serves as an integrative stress sensor, linking environmental cues such as iron deficiency, oxidative stress and chronic inflammation to RB‐mediated cell cycle arrest and cellular senescence. In support of this view, reduced proliferation potential and senescence phenotypes have been reported in MuSC isolated from patients of COPD [34], CHF [35] and CKD [36]. Notably, during cellular senescence, Rb promotes senescence‐associated epigenetic silencing—a function distinct from its classical role in E2F sequestration [20]. Similar epigenetic changes were observed in HIF‐2α OE myoblasts, suggesting that HIF‐2α OE may induce a senescent‐like state. Acute Rb depletion can reverse senescence [37], suggesting that fine‐tuning HIF‐2α activity may restore the balance between proliferation and senescence. In regenerative muscle, pharmacological inhibition of HIF‐2α or Rb enhances MuSC expansion and repair outcomes [15, 16, 28]. Interestingly, while Rb ablation impairs myogenic differentiation, HIF‐2α ablation does not [15, 28], suggesting that HIF‐2α's impact on Rb transactivation is conditional and likely stress‐dependent. This supports the idea that HIF‐2α inhibition may be more effective than direct Rb manipulation for sarcopenia treatment in chronic disease and aging.

The remarkable ability of PT2385 to rescue muscle regeneration in iron‐deficient mice—without replenishing iron levels—highlights the pivotal role of HIF‐2α in driving MuSC proliferation defects under iron deficiency. Given that proliferating MuSC primarily rely on glycolysis for ATP production, they are relatively insensitive to iron‐dependent mitochondrial dysfunction [38]. Thus, by inhibiting the HIF‐2α–Rb axis, PT2385 directly alleviates the proliferation block, allowing MuSC to regain regenerative potential without necessitating an intermediate increase in iron availability.

Additionally, PT2385 may indirectly improve muscle regeneration by altering local iron availability via macrophage‐mediated mechanisms. Macrophages play a critical role in iron recycling and redistribution during muscle repair [11]. Under inflammatory conditions, pro‐inflammatory cytokines such as TNF‐α, IL‐1β, IL‐10 and IFN‐γ promote iron acquisition within macrophages by increasing iron uptake (via TfRc, DMT1 or lactoferrin/lipocalin2 scavenging) and decreasing iron export (via suppression of Ferroportin expression) [39]. This inflammation‐driven iron sequestration within macrophages and shift in iron availability contribute significantly to functional iron deficiency. HIF‐2α^+^ macrophages are enriched in inflamed tissues [33, 40]. Given that HIF‐2α promotes TNF‐α and IFN‐γ production [40], PT2385‐mediated HIF‐2α inhibition may reduce iron sequestration in macrophages, thereby improving iron delivery to MuSC. This hypothesis merits further exploration.

Chronic diseases such as COPD, CHF, CKD, cancer cachexia and aging are often characterized by low‐grade systemic inflammation, with elevated IL‐1β and TNF‐α. Functional iron deficiency is more prevalent than absolution iron deficiency in these conditions. Targeting HIF‐2α may offer an alternative therapeutic approach to counteract muscle wasting in chronic diseases.

## Funding

This work was supported by the National Institutes of Health (NIH), National Institute of Arthritis and Musculoskeletal and Skin Diseases (NIAMS) under grant number R01AR070178.

## Conflicts of Interest

The authors declare no conflicts of interest. A.Y. and H.Y. are employed by HAWA Therapeutics LLC, a small business operating in a field related to the subject matter of this manuscript. While all experiments were conducted according to rigorous scientific standards, and while every effort was made to maintain objectivity and transparency, the authors acknowledge that their professional affiliation with HAWA Therapeutics LLC could potentially influence data interpretation or discussion. This statement is provided in the interest of full disclosure and to uphold the credibility and impartiality of the research presented herein.

## Supporting information


**Figure S1:** jcsm70124‐sup‐0001‐Supplementary_Material.docx. **Validation of MuSC activation and assessment of differentiation and cell death under serum starvation conditions. (A)** RT‐qPCR analysis of *Epas1* (encoding HIF‐2α) and *Spry1* expression in quiescent MuSC (FI‐MuSC) and activated MuSC, isolated at day 0 and day 5 after injury, respectively. Data confirm downregulation of quiescence‐associated genes upon activation. **(B)** Immunofluorescence staining for myogenin in C2C12 myoblasts following serum starvation (noncycling), serum refeeding (cycling) or 2‐day differentiation (positive control). Myogenin expression is absent in noncycling and cycling cells, but robust in differentiated cells. **(C)** Propidium iodide (PI) staining to assess membrane integrity in serum‐starved and serum‐refed myoblasts. Triton X‐100–treated cells serve as a positive control for membrane permeabilization. Minimal PI signal in test conditions indicates low cell death.
**Figure S2: Iron depletion does not induce myoblast death or reduce MuSC number in uninjured muscle. (A)** Live cell imaging of C2C12 myoblasts treated with vehicle (control) or 20 μM BPD (Fe^2+^ chelator) for 5 days. Cells were stained with live‐cell viability dyes: Live blue (nuclear) and Live green (cytosolic membrane damage indicator). BPD‐treated cells showed no increase in green signal, indicating no detectable cytotoxicity. Cells treated with detergent Triton X‐100 (positive) serve as a technical control. **(B)** Quantification of Pax7^+^ MuSC per soleus muscle section in IS and ID mice (*n* = 6 per group). Welch's *t* test did not reveal significant difference between groups, indicating that mild iron deficiency does not deplete the MuSC pool in uninjured soleus muscle.
**Figure S3: Characterization of ID and IS mice following 8‐week dietary intervention. (A)** Tibialis anterior (TA) muscle weight at baseline (0 dpi) following 8 weeks of IS or ID chow feeding. Muscle weights were significantly reduced in ID male mice, while female mice showed a nonsignificant trend (*n* = 6 per group). **(B)** Body weight trajectory during the 8‐week feeding period, showing comparable growth in IS and ID groups across sexes (*n* = 6 per group). **(C)** Haemoglobin levels were significantly lower in ID mice of both sexes, confirming the development of iron deficiency. **(D)** Haematocrit levels remained comparable between IS and ID mice, indicating that the model induces iron deficiency without overt anaemia.
**Figure S4: HIF‐2α overexpression mimics the transcriptional effects of iron deficiency. (A)** Immunoblot showing nuclear stabilization of HIF‐2α in C2C12 myoblasts following doxycycline (Dox) treatment in the HIF‐2α overexpression (OE) Tet‐On system. **(B)** Representative images and quantification of EdU incorporation in control and HIF‐2α OE myoblasts with or without Dox. HIF‐2α overexpression significantly reduced the percentage of EdU^+^ proliferating cells. **(C)** Gene expression scatter plot from RNA‐seq comparing wide‐type (WT) and HIF‐2α OE myoblasts. Each point represents an individual gene, plotted as log_2_‐transformed expression levels in WT control (x‐axis) and HIF‐2α OE (y‐axis) samples. Genes with significantly altered expression (FDR < 0.05) are colour‐coded: red for genes upregulated in HIF‐2α OE, blue for genes upregulated in WT and grey for genes without significant change. *Epas1* and known HIF‐2α target *Vegfa* are labelled. Top 10 upregulated and downregulated protein‐coding genes in HIF‐2α OE myoblasts are listed. **(D)** RT‐qPCR analysis of E2F targets *Ccna2* and *Ccnb1* in control, BPD‐treated and BPD + PT2385 co‐treated myoblasts. PT2385 partially rescues the repression of E2F targets under iron chelation.
**Figure S5: HIF‐2α promotes Rb1 induction. (A)** RT‐qPCR of *Rb1* mRNA showing upregulation in BPD‐treated myoblasts. **(B)** Diagram of the *Rb1* promoter region showing the location of a conserved hypoxia response element (HRE) and primers used for ChIP‐qPCR. **(C)** ChIP‐qPCR confirming HIF‐2α enrichment at promoter regions of known targets *Vegfa* and *Cav1*, validating the specificity of HIF‐2α chromatin association. **(D)** ChIP‐qPCR analysis of H3K27ac enrichment at the *Vegfa* promoter in WT and HIF‐2α OE myoblasts, confirming transcriptional activation.
**Figure S6: PT2385 maintains MuSC self‐renewal capacity during regeneration under iron deficiency. (A) Representative immunofluorescence** images showing Pax7^+^ MuSC (red), Laminin B2 (green, myofibre membrane) and DAPI (blue, nuclei) in TA muscle cross‐sections at 30 dpi from iron‐deficient (ID) mice treated with DMSO or PT2385. Quantification of Pax7^+^ MuSC per cross‐section is shown (*n* = 3 mice per group).

## References

[1] M. Dziegala , K. Josiak , M. Kasztura , et al., “Iron Deficiency as Energetic Insult to Skeletal Muscle in Chronic Diseases,” Journal of Cachexia, Sarcopenia and Muscle 9 (2018): 802–815. 30178922 10.1002/jcsm.12314 PMC6204587

[2] A. H. Nickol , M. C. Frise , H. Y. Cheng , et al., “A Cross‐Sectional Study of the Prevalence and Associations of Iron Deficiency in a Cohort of Patients With Chronic Obstructive Pulmonary Disease,” BMJ Open 5 (2015): e007911. 10.1136/bmjopen-2015-007911 PMC4499677 26150144

[3] I. T. Klip , J. Comin‐Colet , A. A. Voors , et al., “Iron Deficiency in Chronic Heart Failure: An International Pooled Analysis,” American Heart Journal 165 (2013): 575–582.e3. 23537975 10.1016/j.ahj.2013.01.017

[4] D. Hain , D. Bednarski , M. Cahill , et al., “Iron‐Deficiency Anemia in CKD: A Narrative Review for the Kidney Care Team,” Kidney Med 5 (2023): 100677. 37415621 10.1016/j.xkme.2023.100677 PMC10319843

[5] H. Ludwig , E. Muldur , G. Endler , and W. Hubl , “Prevalence of Iron Deficiency Across Different Tumors and Its Association With Poor Performance Status, Disease Status and Anemia,” Annals of Oncology 24 (2013): 1886–1892. 23567147 10.1093/annonc/mdt118 PMC3690908

[6] J. S. J. Vinke , A. R. Gorter , M. F. Eisenga , et al., “Iron Deficiency Is Related to Lower Muscle Mass in Community‐Dwelling Individuals and Impairs Myoblast Proliferation,” Journal of Cachexia, Sarcopenia and Muscle 14 (2023): 1865–1879. 37386912 10.1002/jcsm.13277 PMC10401536

[7] M. D. Cappellini , J. Comin‐Colet , A. de Francisco , et al., “Iron Deficiency Across Chronic Inflammatory Conditions: International Expert Opinion on Definition, Diagnosis, and Management,” American Journal of Hematology 92 (2017): 1068–1078. 28612425 10.1002/ajh.24820 PMC5599965

[8] M. Huang , B. Xu , Y. Xu , et al., “Serum Iron Level Is Independently Associated With Sarcopenia: A Retrospective Study,” Scientific Reports 14 (2024): 10554. 38719903 10.1038/s41598-024-61429-0 PMC11078979

[9] H. Yin , F. Price , and M. A. Rudnicki , “Satellite Cells and the Muscle Stem Cell Niche,” Physiological Reviews 93 (2013): 23–67. 23303905 10.1152/physrev.00043.2011 PMC4073943

[10] Y. Che , J. Li , P. Wang , et al., “Iron Deficiency‐Induced Ferritinophagy Impairs Skeletal Muscle Regeneration Through RNF20‐Mediated H2Bub1 Modification,” Science Advances 9 (2023): eadf4345. 37976359 10.1126/sciadv.adf4345 PMC10656073

[11] G. Corna , I. Caserta , A. Monno , et al., “The Repair of Skeletal Muscle Requires Iron Recycling Through Macrophage Ferroportin,” Journal of Immunology 197 (2016): 1914–1925. 10.4049/jimmunol.1501417 27465531

[12] K. Pantopoulos , S. K. Porwal , A. Tartakoff , and L. Devireddy , “Mechanisms of Mammalian Iron Homeostasis,” Biochemistry 51 (2012): 5705–5724. 22703180 10.1021/bi300752r PMC3572738

[13] E. Patino , D. Bhatia , S. Z. Vance , et al., “Iron Therapy Mitigates Chronic Kidney Disease Progression by Regulating Intracellular Iron Status of Kidney Macrophages,” JCI Insight 8, no. 1 (2023): e159235. 36394951 10.1172/jci.insight.159235 PMC9870080

[14] S. Lakhal‐Littleton , Cardiomyocyte Hepcidin: From Intracellular Iron Homeostasis to Physiological Function. Vitamins and Hormones 110, (2019) 189–200. 30798812 10.1016/bs.vh.2019.01.009

[15] L. Xie , A. Yin , A. S. Nichenko , A. M. Beedle , J. A. Call , and H. Yin , “Transient HIF2A Inhibition Promotes Satellite Cell Proliferation and Muscle Regeneration,” Journal of Clinical Investigation 128 (2018): 2339–2355. 29533927 10.1172/JCI96208 PMC5983316

[16] A. Yin , W. Fu , A. Elengickal , et al., “Chronic Hypoxia Impairs Skeletal Muscle Repair via HIF‐2alpha Stabilization,” Journal of Cachexia, Sarcopenia and Muscle 15 (2024): 631–645. 38333911 10.1002/jcsm.13436 PMC10995261

[17] T. Chaillou and J. T. Lanner , “Regulation of Myogenesis and Skeletal Muscle Regeneration: Effects of Oxygen Levels on Satellite Cell Activity,” FASEB Journal 30 (2016): 3929–3941. 27601440 10.1096/fj.201600757R

[18] A. Jaitovich , “Impaired Regenerative Capacity Contributes to Skeletal Muscle Dysfunction in Chronic Obstructive Pulmonary Disease,” American Journal of Physiology. Cell Physiology 323 (2022): C974–C989. 35993519 10.1152/ajpcell.00292.2022 PMC9484993

[19] A. Brehm , E. A. Miska , D. J. McCance , J. L. Reid , A. J. Bannister , and T. Kouzarides , “Retinoblastoma Protein Recruits Histone Deacetylase to Repress Transcription,” Nature 391 (1998): 597–601. 9468139 10.1038/35404

[20] M. Narita , S. Nunez , E. Heard , et al., “Rb‐Mediated Heterochromatin Formation and Silencing of E2F target Genes During Cellular Senescence,” Cell 113 (2003): 703–716. 12809602 10.1016/s0092-8674(03)00401-x

[21] C. Peyssonnaux , A. S. Zinkernagel , R. A. Schuepbach , et al., “Regulation of Iron Homeostasis by the Hypoxia‐Inducible Transcription Factors (HIFs),” Journal of Clinical Investigation 117 (2007): 1926–1932. 17557118 10.1172/JCI31370 PMC1884690

[22] D. R. Mole , “Iron Homeostasis and Its Interaction With Prolyl Hydroxylases,” Antioxidants & Redox Signaling 12 (2010): 445–458. 19650690 10.1089/ars.2009.2790

[23] M. R. Davis , K. M. Shawron , E. Rendina , et al., “Hypoxia Inducible Factor‐2 Alpha Is Translationally Repressed in Response to Dietary Iron Deficiency in Sprague‐Dawley Rats,” Journal of Nutrition 141 (2011): 1590–1596. 21753061 10.3945/jn.111.144105 PMC3735917

[24] Z. Feng , X. Zou , Y. Chen , H. Wang , Y. Duan , and R. K. Bruick , “Modulation of HIF‐2alpha PAS‐B Domain Contributes to Physiological Responses,” Proceedings of the National Academy of Sciences of the United States of America 115 (2018): 13240–13245. 30523118 10.1073/pnas.1810897115 PMC6310796

[25] Y. M. Shah , T. Matsubara , S. Ito , S. H. Yim , and F. J. Gonzalez , “Intestinal Hypoxia‐Inducible Transcription Factors Are Essential for Iron Absorption Following Iron Deficiency,” Cell Metabolism 9 (2009): 152–164. 19147412 10.1016/j.cmet.2008.12.012 PMC2659630

[26] V. H. Haase , “Regulation of Erythropoiesis by Hypoxia‐Inducible Factors,” Blood Reviews 27 (2013): 41–53. 23291219 10.1016/j.blre.2012.12.003 PMC3731139

[27] L. Hagler , E. W. Askew , J. R. Neville , P. W. Mellick , R. I. Coppes, Jr. , and J. F. Lowder, Jr. , “Influence of Dietary Iron Deficiency on Hemoglobin, Myoglobin, Their Respective Reductases, and Skeletal Muscle Mitochondrial Respiration,” American Journal of Clinical Nutrition 34 (1981): 2169–2177. 6271003 10.1093/ajcn/34.10.2169

[28] T. Hosoyama , K. Nishijo , S. I. Prajapati , G. Li , and C. Keller , “Rb1 Gene Inactivation Expands Satellite Cell and Postnatal Myoblast Pools,” Journal of Biological Chemistry 286 (2011): 19556–19564. 21478154 10.1074/jbc.M111.229542 PMC3103335

[29] I. J. Cho , P. P. Lui , J. Obajdin , et al., “Mechanisms, Hallmarks, and Implications of Stem Cell Quiescence,” Stem Cell Reports 12 (2019): 1190–1200. 31189093 10.1016/j.stemcr.2019.05.012 PMC6565921

[30] A. Mohyeldin , T. Garzon‐Muvdi , and A. Quinones‐Hinojosa , “Oxygen in Stem Cell Biology: A Critical Component of the Stem Cell Niche,” Cell Stem Cell 7 (2010): 150–161. 20682444 10.1016/j.stem.2010.07.007

[31] Y. Pan , K. D. Mansfield , C. C. Bertozzi , et al., “Multiple Factors Affecting Cellular Redox Status and Energy Metabolism Modulate Hypoxia‐Inducible Factor Prolyl Hydroxylase Activity In Vivo and In Vitro,” Molecular and Cellular Biology 27 (2007): 912–925. 17101781 10.1128/MCB.01223-06 PMC1800695

[32] B. K. Nayak , D. Feliers , S. Sudarshan , et al., “Stabilization of HIF‐2alpha Through Redox Regulation of mTORC2 Activation and Initiation of mRNA Translation,” Oncogene 32 (2013): 3147–3155. 22869144 10.1038/onc.2012.333 PMC3696051

[33] N. Takeda , E. L. O'Dea , A. Doedens , et al., “Differential Activation and Antagonistic Function of HIF‐Alpha Isoforms in Macrophages Are Essential for NO Homeostasis,” Genes & Development 24 (2010): 491–501. 20194441 10.1101/gad.1881410 PMC2827844

[34] M. E. Theriault , M. E. Pare , F. Maltais , and R. Debigare , “Satellite Cells Senescence in Limb Muscle of Severe Patients With COPD,” PLoS ONE 7 (2012): e39124. 22720047 10.1371/journal.pone.0039124 PMC3374758

[35] T. Sente , A. M. Van Berendoncks , A. I. Jonckheere , et al., “Primary Skeletal Muscle Myoblasts From Chronic Heart Failure Patients Exhibit Loss of Anti‐Inflammatory and Proliferative Activity,” BMC Cardiovascular Disorders 16 (2016): 107. 27228977 10.1186/s12872-016-0278-3 PMC4880810

[36] L. Zhang , X. H. Wang , H. Wang , J. Du , and W. E. Mitch , “Satellite Cell Dysfunction and Impaired IGF‐1 Signaling Cause CKD‐Induced Muscle Atrophy,” J Am Soc Nephrol 21 (2010): 419–427. 20056750 10.1681/ASN.2009060571 PMC2831855

[37] J. Sage , A. L. Miller , P. A. Perez‐Mancera , J. M. Wysocki , and T. Jacks , “Acute Mutation of Retinoblastoma Gene Function Is Sufficient for Cell Cycle Re‐Entry,” Nature 424 (2003): 223–228. 12853964 10.1038/nature01764

[38] J. G. Ryall , S. Dell'Orso , A. Derfoul , et al., “The NAD(+)‐Dependent SIRT1 Deacetylase Translates a Metabolic Switch into Regulatory Epigenetics in Skeletal Muscle Stem Cells,” Cell Stem Cell 16 (2015): 171–183. 25600643 10.1016/j.stem.2014.12.004 PMC4320668

[39] G. Weiss , T. Ganz , and L. T. Goodnough , “Anemia of Inflammation,” Blood 133 (2019): 40–50. 30401705 10.1182/blood-2018-06-856500 PMC6536698

[40] H. Z. Imtiyaz , E. P. Williams , M. M. Hickey , et al., “Hypoxia‐Inducible Factor 2alpha Regulates Macrophage Function in Mouse Models of Acute and Tumor Inflammation,” Journal of Clinical Investigation 120 (2010): 2699–2714. 20644254 10.1172/JCI39506 PMC2912179

