# Supplementary material for: Iron Deficiency Impairs Muscle Stem Cell Proliferation and Skeletal Muscle Regeneration via HIF‐2α Stabilization
Source: J Cachexia Sarcopenia Muscle. 2025 Nov 25;16(6):e70124. doi: 10.1002/jcsm.70124 (PMC12647922; doi:10.1002/jcsm.70124)
Supplement: Supplementary file 1 — Figure S1: jcsm70124‐sup‐0001‐Supplementary_Material.docx. Validation of MuSC activation and assessment of differentiation and cell death under serum starvation conditions. (A) RT‐qPCR analysis of Epas1 (encoding HIF‐2α) and Spry1 expression in quiescent MuSC (FI‐MuSC) and activated MuSC, isolated at day 0 and day 5 after injury, respectively. Data confirm downregulation of quiescence‐associated genes upon activation. (B) Immunofluorescence staining for myogenin in C2C12 myoblasts following serum starvation (noncycling), serum refeeding (cycling) or 2‐day differentiation (positive control). Myogenin expression is absent in noncycling and cycling cells, but robust in differentiated cells. (C) Propidium iodide (PI) staining to assess membrane integrity in serum‐starved and serum‐refed myoblasts. Triton X‐100–treated cells serve as a positive control for membrane permeabilization. Minimal PI signal in test conditions indicates low cell death. Figure S2: Iron depletion does not induce myoblast death or reduce MuSC number in uninjured muscle. (A) Live cell imaging of C2C12 myoblasts treated with vehicle (control) or 20 μM BPD (Fe2+ chelator) for 5 days. Cells were stained with live‐cell viability dyes: Live blue (nuclear) and Live green (cytosolic membrane damage indicator). BPD‐treated cells showed no increase in green signal, indicating no detectable cytotoxicity. Cells treated with detergent Triton X‐100 (positive) serve as a technical control. (B) Quantification of Pax7+ MuSC per soleus muscle section in IS and ID mice (n = 6 per group). Welch's t test did not reveal significant difference between groups, indicating that mild iron deficiency does not deplete the MuSC pool in uninjured soleus muscle. Figure S3: Characterization of ID and IS mice following 8‐week dietary intervention. (A) Tibialis anterior (TA) muscle weight at baseline (0 dpi) following 8 weeks of IS or ID chow feeding. Muscle weights were significantly reduced in ID male mice, while female mice showe [file JCSM-16-e70124-s001.docx]

**SUPPLEMENTARY METHODS**

**Inducible HIF-2α Overexpression Construction**

A stabilized form of human HIF-2α was cloned from pcDNA3 HIF-2α MYC TM (P405A/P530V/N851A, Addgene #44027) into Tet-on inducible expression system pCW57 (Addgene #71783) for lentivirus packaging. C2C12 inducible HIF-2α overexpression cell line was constructed by pCW57-HIF-2α TM OE lentivirus transduction and neomycin selection. HIF-2α overexpression was induced by 250 ng/mL doxycycline in culture media at the stated time.

**siRNA Treatment on HIF-2α OE Myoblasts**

HIF-2α OE myoblasts were treated with siRNA smart pool against *Rb1* (Santa Cruz Biotechnology, sc-29469) or control siRNA (Santa Cruz Biotechnology, sc-36869). Transfection was performed using Lipofectamine RNAiMAX (Invitrogen, Carlsbad, CA, USA) according to the manufacturer’s instructions.

**Flow Cytometry for CD71**

Cells were resuspended with flow cytometry buffer consisting of 1% fetal bovine serum (FBS), 1 mM EDTA, and 25 mM HEPES in PBS, and incubated with anti-CD71 antibody (BioLegend #113805) on ice for 30 minutes. Following incubation, cells were washed twice and analyzed using CytoFlex Cytometer (Beckman Coulter). Data were processed and analyzed using FlowJo v.10.

**Western Blotting**

Protein lysates were prepared by lysing cells/tissues in RIPA buffer supplemented with a proteinase inhibitor cocktail (1x). Protein concentration was quantified by BCA Protein Assays (ThermoFisherScientific). The membrane was blocked with 5% non-fat milk/TBST and probed with primary antibodies: anti-HIF-2α (1:1,000, Novus #NB100-122), anti-TrR1 (1:1,000, Abcam #Ab84036), and anti-alpha-Tubulin (1:5,000; Sigma #T6199), incubated with ECL reagents (Santa Cruz) and exposed to X-ray films.

**Chromatin Immunoprecipitation (ChIP) and qPCR**

PFA-fixed cells were resuspended in 200 μL lysis buffer and sonicated at 4°C in a Bioruptor® Pico sonicator (Diagenode). The sheared chromatin (~300 bp) was diluted in IP dilution buffer (50 mM HEPES-KOH pH7.5, 140 mM NaCl, 1 mM EDTA, 1% Triton X-100, 0.1% sodium deoxycholate, 0.1% SDS) and incubated with 2 μg HIF-2α antibody (Novus #NB100-122) or rabbit IgG (Santa Cruz) at 4°C overnight. Protein A Dynabeads^®^ (30 μL 50% slurry) were added to the chromatin/antibody mix and incubated at 4°C for 4 hrs. All ChIP beads were sequentially washed with low salt, high salt, lithium, and TE buffer for 2 times/each and reverse-crosslinked with 1M NaCl for 6 hrs at 65°C. Samples were digested with 10 μg RNase A (37°C, 0.5 hr) and 20 μg Proteinase K (55°C, overnight). Immunoprecipitated genomic DNA was purified. The following primers were used to quantify relative enrichment levels at HRE-flanking regions in the promoter of *ACE* in the mouse genome:

Rb1_HRE_S: 5’-GGCCGGCAAAACAAAAACA-3’

Rb1_HRE_AS: 5’-TTACCGGGGTGGGACC-3’

**SUPPLEMENTARY FIGURES**


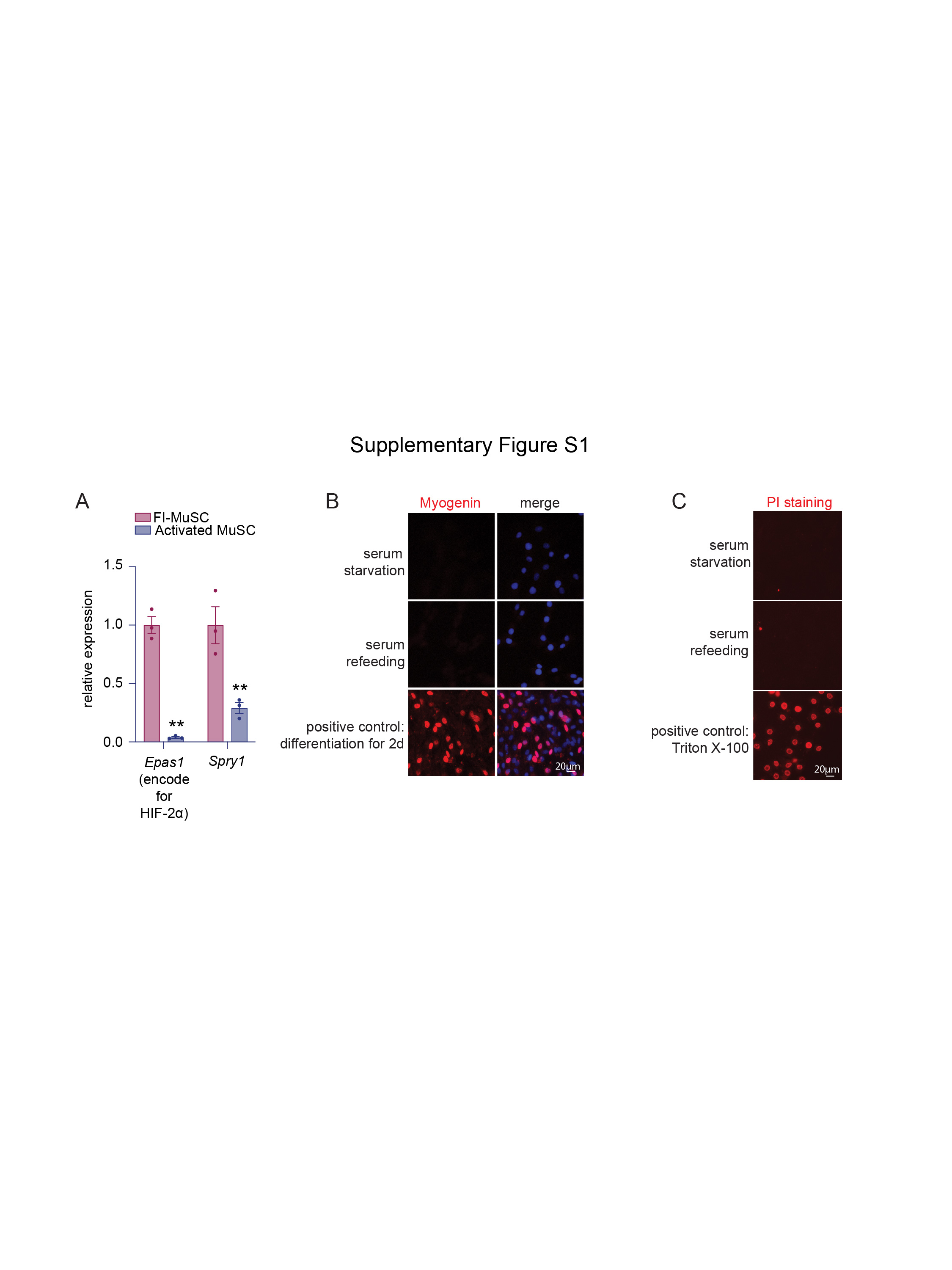


**Figure S1 (associated with Figure 1)**

**Supplementary Figure S1. Validation of MuSC activation and assessment of differentiation and cell death under serum starvation conditions.**
**(A)** RT-qPCR analysis of Epas1 (encoding HIF-2α) and Spry1 expression in quiescent MuSC (FI-MuSC) and activated MuSC, isolated at day 0 and day 5 post-injury, respectively. Data confirm downregulation of quiescence-associated genes upon activation.
**(B)** Immunofluorescence staining for myogenin in C2C12 myoblasts following serum starvation (non-cycling), serum refeeding (cycling), or 2-day differentiation (positive control). Myogenin expression is absent in non-cycling and cycling cells, but robust in differentiated cells.
**(C)** Propidium iodide (PI) staining to assess membrane integrity in serum-starved and serum-refed myoblasts. Triton X-100–treated cells serve as a positive control for membrane permeabilization. Minimal PI signal in test conditions indicates low cell death.


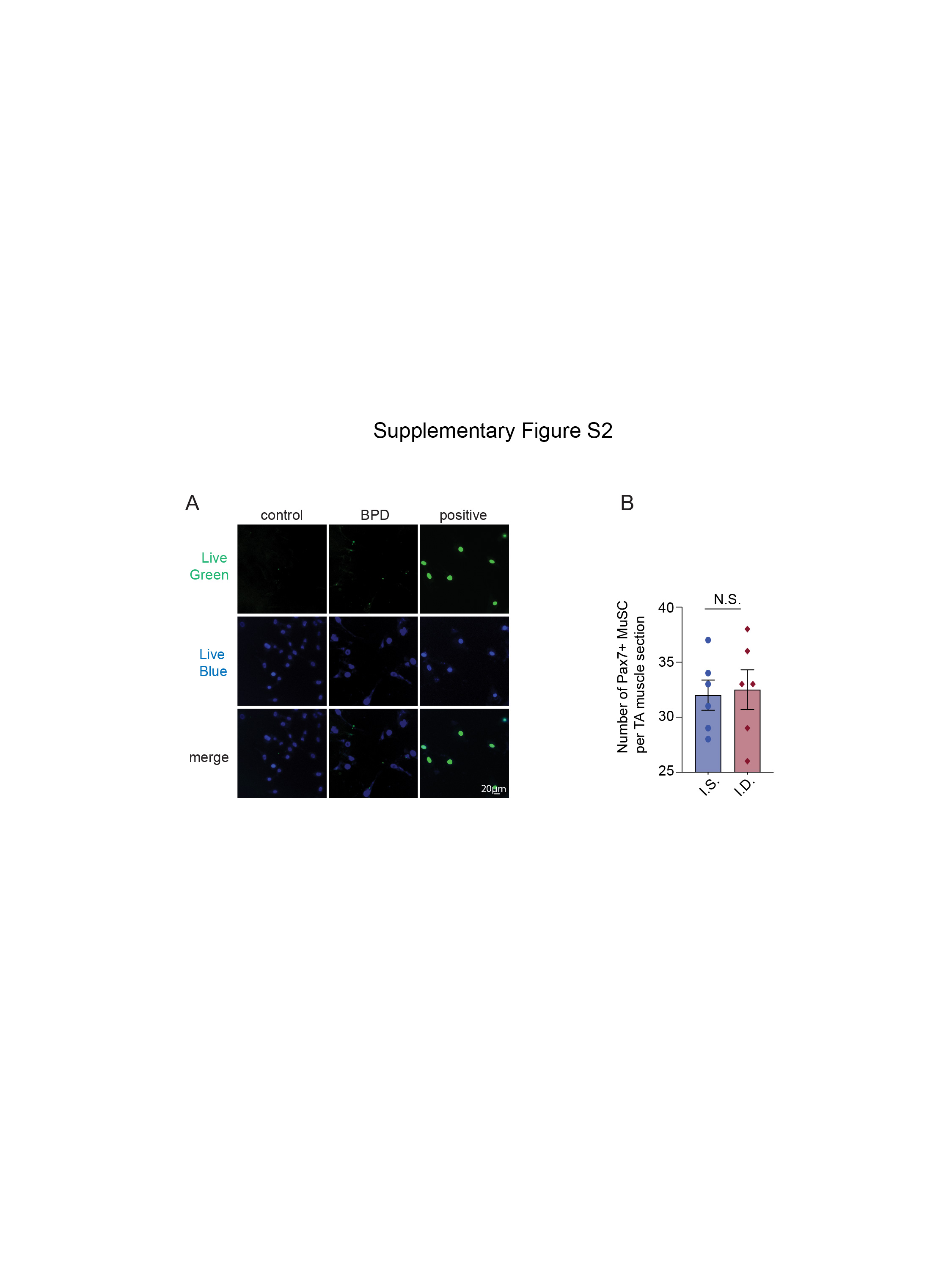


**Figure S2 (associated with Figure 2)**

**Supplementary Figure S2. Iron depletion does not induce myoblast death or reduce MuSC number in uninjured muscle.**
**(A)** Live cell imaging of C2C12 myoblasts treated with vehicle (control) or 20 μM BPD (Fe^2+^ chelator) for 5 days. Cells were stained with live-cell viability dyes: Live blue (nuclear) and Live green (cytosolic membrane damage indicator). BPD-treated cells showed no increase in green signal, indicating no detectable cytotoxicity. Cells treated with detergent Triton X-100 (positive) serve as a technical control.
**(B)** Quantification of Pax7⁺ MuSC per soleus muscle section in IS and ID mice (n = 6 per group). Welch's *t*-test did not reveal significant difference between groups, indicating that mild iron deficiency does not deplete the MuSC pool in uninjured soleus muscle.


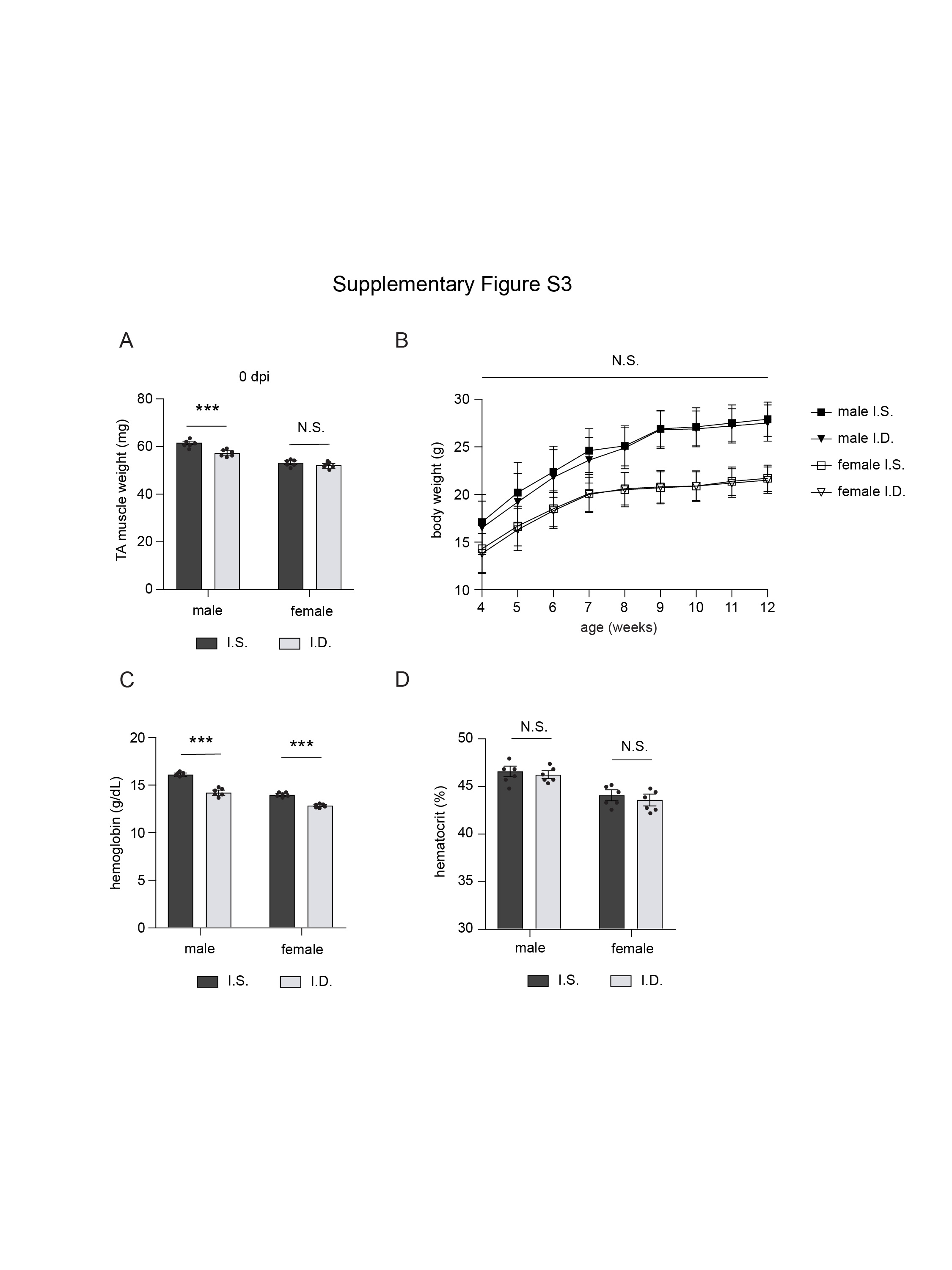


**Figure S3 (associated with Figure 3)**

**Supplementary Figure S3. Characterization of ID and IS mice following 8-week dietary intervention.**
**(A)** Tibialis anterior (TA) muscle weight at baseline (0 dpi) following 8 weeks of IS or ID chow feeding. Muscle weights were significantly reduced in ID male mice, while female mice showed a non-significant trend (n = 6 per group).
**(B)** Body weight trajectory during the 8-week feeding period, showing comparable growth in IS and ID groups across sexes (n = 6 per group).
**(C)** Hemoglobin levels were significantly lower in ID mice of both sexes, confirming the development of iron deficiency.
**(D)** Hematocrit levels remained comparable between IS and ID mice, indicating that the model induces iron deficiency without overt anemia.


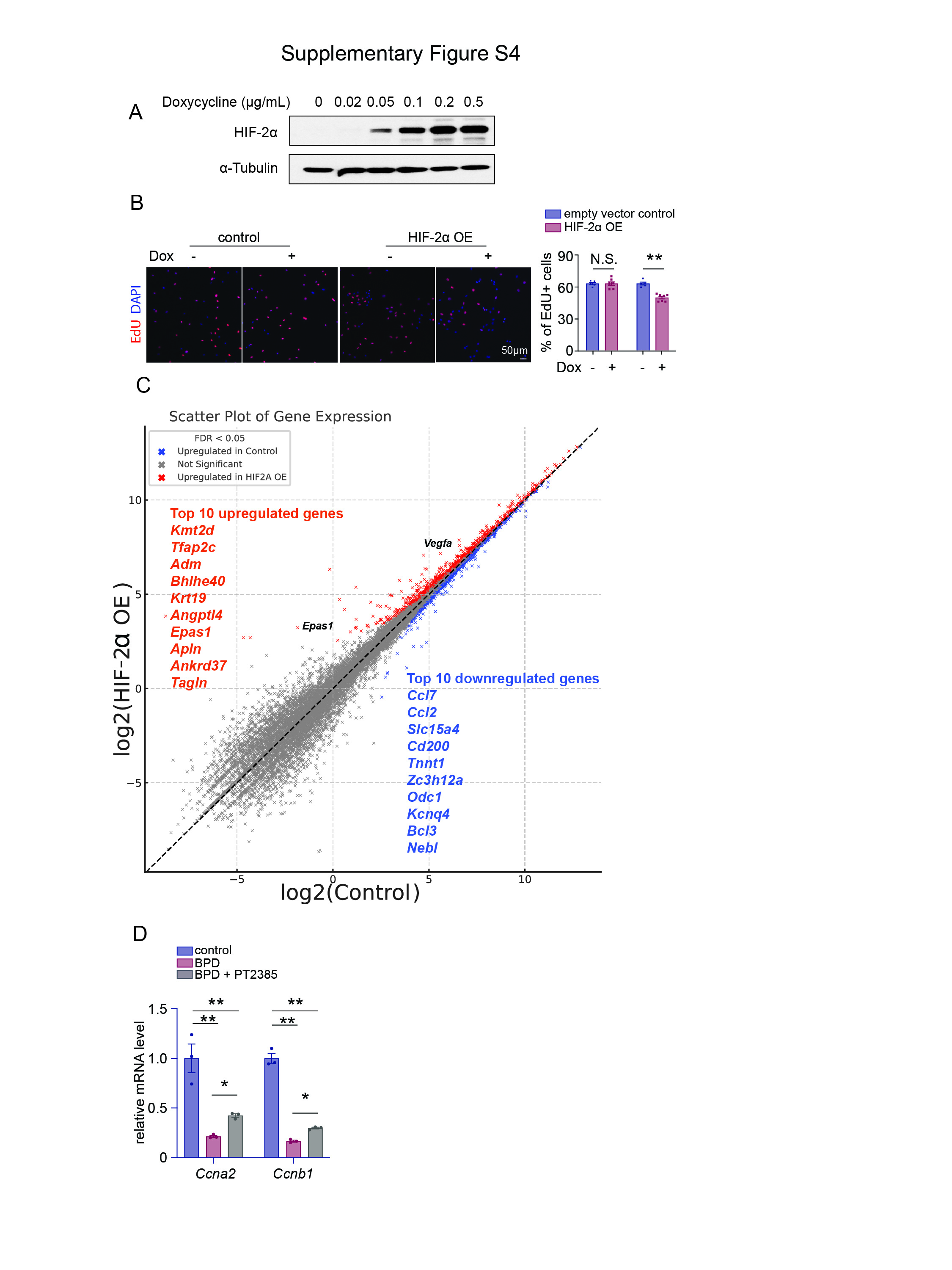


**Figure S4 (associated with Figure 5)**

**Supplementary Figure S4. HIF-2α overexpression mimics the transcriptional effects of iron deficiency.**
**(A)** Immunoblot showing nuclear stabilization of HIF-2α in C2C12 myoblasts following doxycycline (Dox) treatment in the HIF-2α overexpression (OE) Tet-On system.
**(B)** Representative images and quantification of EdU incorporation in control and HIF-2α OE myoblasts with or without Dox. HIF-2α overexpression significantly reduced the percentage of EdU⁺ proliferating cells.
**(C)** Gene expression scatter plot from RNA-seq comparing wide-type (WT) and HIF-2α OE myoblasts. Each point represents an individual gene, plotted as log_2_-transformed expression levels in WT control (x-axis) and HIF-2α OE (y-axis) samples. Genes with significantly altered expression (FDR < 0.05) are color-coded: red for genes upregulated in HIF-2α OE, blue for genes upregulated in WT, and gray for genes without significant change. *Epas1* and known HIF-2α target *Vegfa* are labeled. Top 10 upregulated and downregulated protein-coding genes in HIF-2α OE myoblasts are listed.
**(D)** RT-qPCR analysis of E2F targets *Ccna2* and *Ccnb1* in control, BPD-treated, and BPD+PT2385 co-treated myoblasts. PT2385 partially rescues the repression of E2F targets under iron chelation.

**
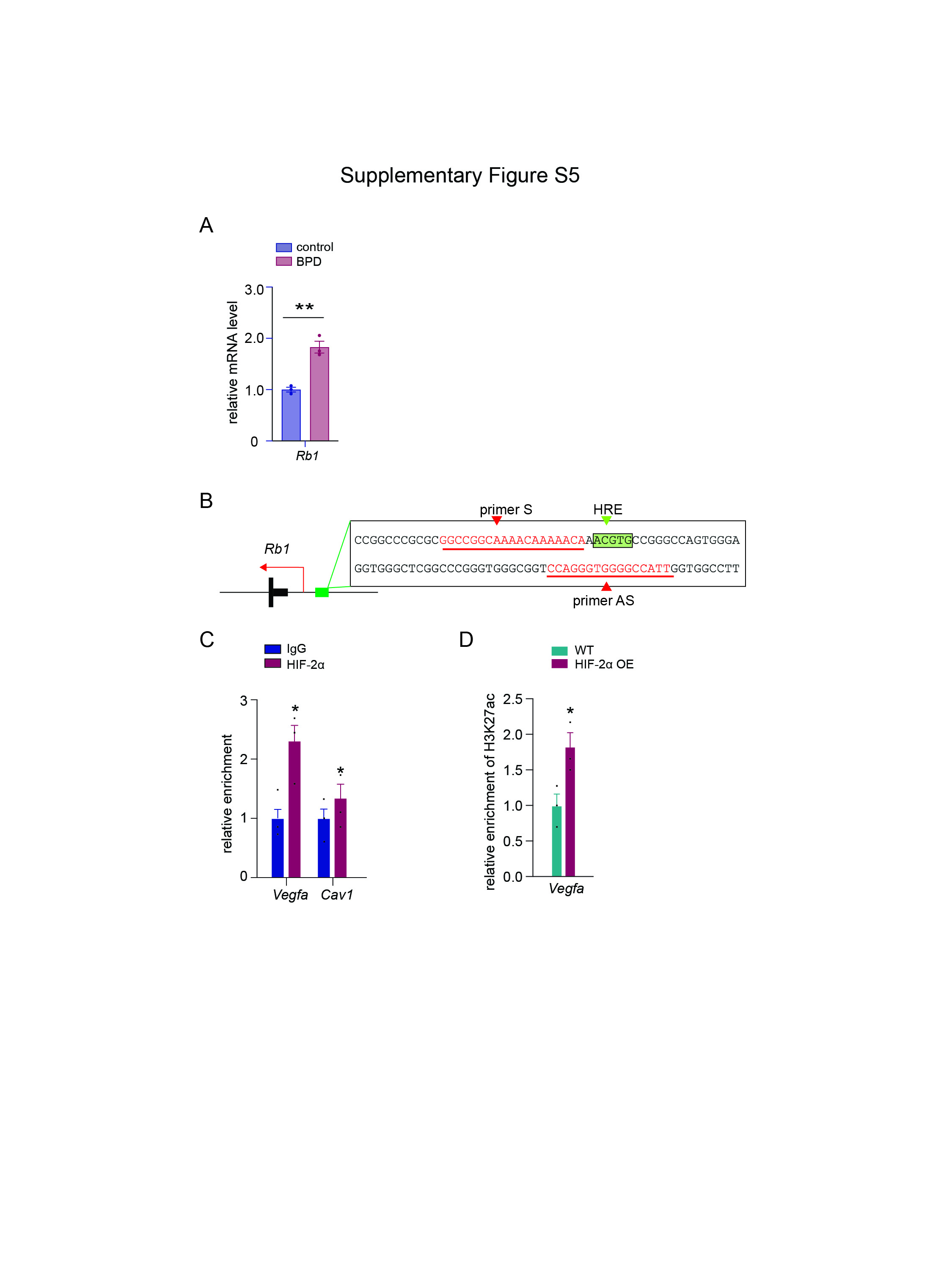
**

**Figure S5 (associated with Figure 6)**

**Supplementary Figure S5. HIF-2α promotes *Rb1* induction.**
**(A)** RT-qPCR of *Rb1* mRNA showing upregulation in BPD-treated myoblasts.

**(B)** Diagram of the *Rb1* promoter region showing the location of a conserved hypoxia response element (HRE) and primers used for ChIP-qPCR.
**(C)** ChIP-qPCR confirming HIF-2α enrichment at promoter regions of known targets *Vegfa* and *Cav1*, validating the specificity of HIF-2α chromatin association.
**(D)** ChIP-qPCR analysis of H3K27ac enrichment at the *Vegfa* promoter in WT and HIF-2α OE myoblasts, confirming transcriptional activation.


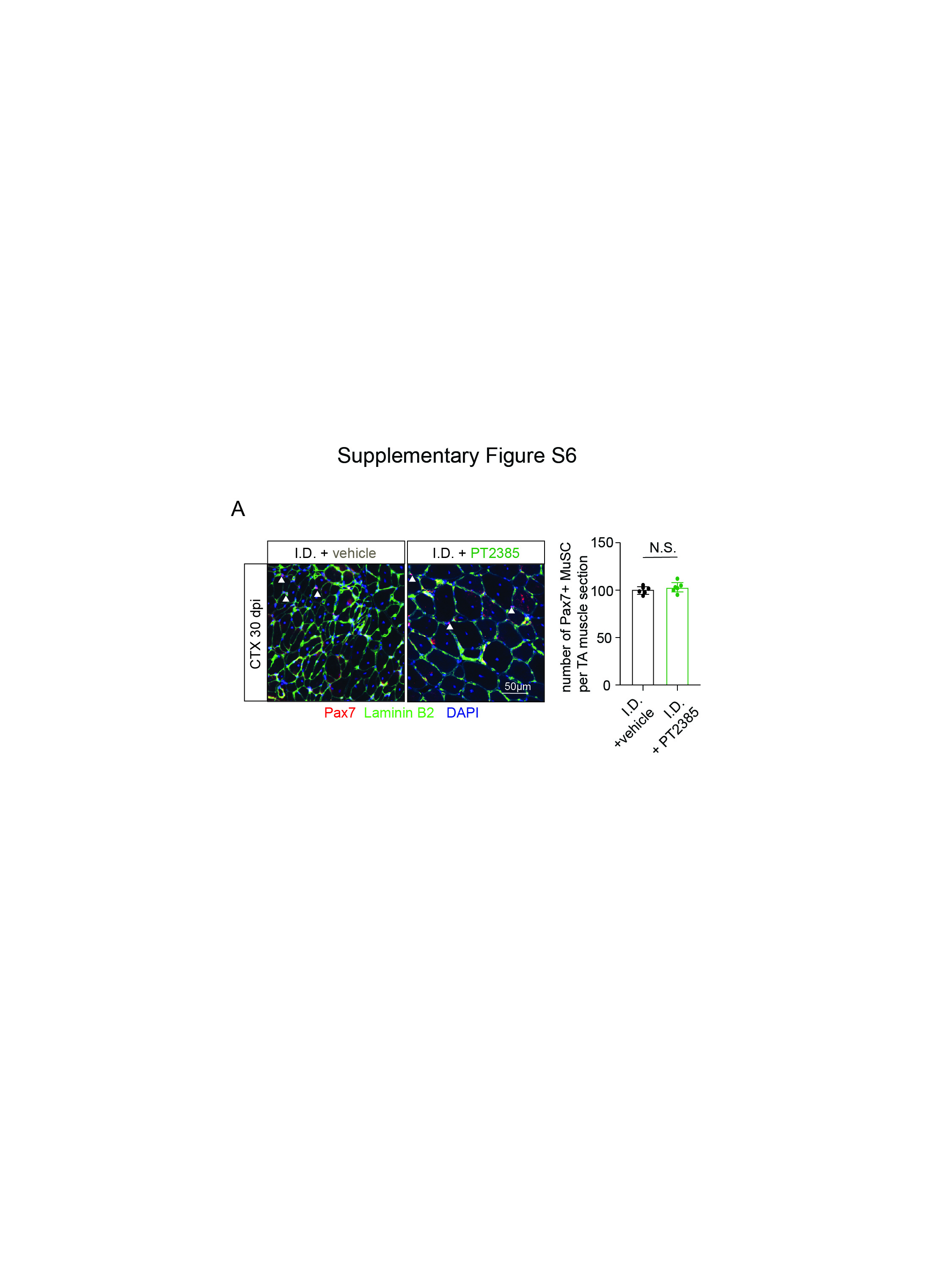


**Figure S6 (associated with Figure 7)**

**Supplementary Figure S6. PT2385 maintains MuSC self-renewal capacity during regeneration under iron deficiency.**

**(A) Representative immunofluorescence** images showing Pax7⁺ MuSC (red), Laminin B2 (green, myofiber membrane), and DAPI (blue, nuclei) in TA muscle cross-sections at 30 dpi from iron-deficient (ID) mice treated with DMSO or PT2385. Quantification of Pax7⁺ MuSC per cross-section is shown (N = 3 mice per group).

**SUPPLEMENTARY REFERENCES**

(1, 2)

S1. Epsztejn, S., Kakhlon, O., Glickstein, H., Breuer, W., and Cabantchik, I. (1997) Fluorescence analysis of the labile iron pool of mammalian cells. *Anal Biochem* 248, 31-40

S2. Prabhune, N. M., Ameen, B., and Prabhu, S. (2024) Therapeutic potential of synthetic and natural iron chelators against ferroptosis. *Naunyn Schmiedebergs Arch Pharmacol*
